# Preparation of new surface coating based on modified oil-based polymers blended with ZnO and CuZnO NPs for steel protection

**DOI:** 10.1038/s41598-023-34085-z

**Published:** 2023-05-04

**Authors:** Roma. G. Elfadel, Hala M. Refat, H. Abd El-Wahab, Salem S. Salem, M. E. Owda, M. A. M. Abdel Reheim

**Affiliations:** 1 Department of Chemistry, Faculty of Science, Arish University, Arish, 45511 Egypt; 2 Department of Chemistry, Faculty of Science, Al-Azhar University, Cairo, 11884 Egypt; 3 Botany and Microbiology Department, Faculty of Science, Al-Azhar University, Cairo, 11884 Egypt

**Keywords:** Chemistry, Nanoscience and technology

## Abstract

In our paper, we have synthesized modified PEA and alkyd resin by replacing the new source of polyol (SDEA) which was confirmed by different analyses such as IR, and 1HNMR spectra. A series of conformal, novel, low-cost, and eco-friendly hyperbranched modified alkyd and PEA resins were fabricated with bio ZnO, CuO/ZnO) NPs through an ex-situ method for mechanical and anticorrosive coatings. The synthesized biometal oxides NPs and its composite modified alkyd and PEA were confirmed by FTIR, SEM with EDEX, TEM, and TGA, and can be stably dispersed into modified alkyd and PEA resins at a low weight fraction of 1%. The nanocomposite coating was also subjected to various tests to determine their surface adhesion, which ranged from (4B-5B), physico-mechanical characteristics such as scratch hardness, which improved from < 1.5 to > 2 kg, gloss (100–135) Specific gravity (0.92–0.96) and also chemical resistance test which passed for water, acid, and solvent except alkali, was poor because of the hydrolyzable ester group in the alkyd and PEA resins. The anti-corrosive features of the nanocomposites were investigated through salt spray tests in 5 wt % NaCl. The results indicate that well-dispersed bio ZnO and CuO/ZnO) NPs (1.0%) in the interior of the hyperbranched alkyd and PEA matrix improve the durability and anticorrosive attributes of the composites, such as degree of rusting, which ranged from 5 to 9, blistering size ranged from 6 to 9, and finally, scribe failure, which ranged from 6 to 9 mm. Thus, they exhibit potential applications in eco- friendly surface coatings. The anticorrosion mechanisms of the nanocomposite alkyd and PEA coating were attributed to the synergistic effect of bio ZnO and (CuO/ZnO) NPs and the prepared modified resins are highly rich in nitrogen elements, which might be regarded as a physical barrier layer for steel substrates.

## Introduction

Nanocomposite coatings are a new class of coatings made to provide smart, affordable, and efficient surface coatings with exceptional characteristics for applications such as corrosion resistance, antimicrobial protection, antifogging, and adhesives^1,2^. Nanocomposite coatings are usually preferable to traditional coatings due to their improved morphology and phase-separated nanoscale domains^3^. Nanostructured filler particles are spread throughout a matrix to create a nanocomposite coating. The chemical nature of the phases, their relative proportions, and the size of the filler all affect the properties of nanocomposite coatings^4^. Nanoparticle type, shape, size, surface area, percentage of nanofillers, and interactions between the nanoparticles and polymer matrix all have a significant role in the features of nanocomposite coatings^5,6^. The best corrosion resistance may be achieved by evenly dispersing nanoparticles inside the polymer matrix. There are several production techniques and physical, chemical, and mechanical surface treatments that can be used to accomplish this^7^. The polyesteramide hyperbranched polymer-modified nano clay structure demonstrated reduced water permeability and improved corrosion resistance^8^. By adding nanomaterial fillers to polymer matrices, several properties, including stiffness, strength, corrosion resistance, and water resistance, may be enhanced^9^. A nanostructured chitosan/ZnO coating was demonstrated to prevent corrosion on mild steel, with corrosion resistance increasing as the chitosan/ZnO layer was raised. A chitosan and graphene oxide nanocomposite coating that had been modified by oleic acid increased corrosion resistance by a factor of 100 in NaCl solution. The improvement in corrosion resistance was attributable to the nanocomposite coating, which also reduced hydrophilicity, oxygen permeability, and ion transport^1,10–12^. The most recent advancements in nanotechnology are focused on the design and functionalization of nanoparticles before they are incorporated into nanocomposite coatings, allowing them to provide effective and ongoing corrosion prevention activity^13^. Numerous techniques were suggested for the production of nanocomposite coatings, but reactive magnetron sputtering is the method that is most frequently utilized. It is feasible to prepare nanocomposite coatings that offer surfaces with a higher hardness than what the law of mixing permits (materials with a hardness of at least 80 GPa are called ultra-hard materials)^14^. It has been reported that a novel hybrid nanocomposite was developed to reduce the rate of corrosion that occurs on the surface of mild steel and to improve its mechanical properties. To address this, a hybrid nanocomposite made of Multi-Walled Carbon Nano-Tubes (MWCNTs), Zinc Oxide nanoparticles, and Epoxy resin was used to resist corrosion, which is the main drawback of mild steel^15^. So, the mechanical properties of mild steel mechanical properties are improved. ZnO nanoparticles and MWCNTs are better dispersed using the ultrasonication technique. Application of MWCNTs/ZnO nanoparticles/Epoxy resin enhanced the rate of anticorrosion and mechanical properties^16^. Mechanical characteristic tests of the coating material with nano modification for colored antiskid pavement were evaluated and assessed. The mechanical characteristics of Poly(methyl methacrylate) (PMMA) material were adjusted by adding an montmorillonite (MMT) nano modifying agent to provide a better-colored road anti-skid surface coating substance^17^. Conductive, biocompatible, and intelligent polymer nanocomposite coatings fall within this category. Since the fundamental characteristics of coatings and adhesives are comparable, polymer nanocomposite adhesives are no different^18^. Coatings made of nanocomposite materials exhibit improvements in quality, cost, intelligence, and efficacy. Despite the reality that the other corrosion prevention methods have a great inhibitory property. However, the nanofillers and nanomatrices in the nanocomposite release active ingredients that repair the damaged surface and slow down the corrosion process when the film of metals coated with the nanocomposite suffers damage in a highly corrosive environment^19^. Fumed silica nanoparticle-encrusted modified poly (ester amide urethane) coatings were created. Jatropha oil (JO) was transformed into JO fatty amide (HEJA), which was then modified by a tetra functional carboxylic acid to create poly (diamino cyclohexane ester amide) (PDCEA). This compound was then combined with fumed silica and toluene 2,4-diisocyanate to create a nanocomposite called (PUDCEA), which demonstrated excellent physico-mechanical and anticorrosion properties up to 200 °C, these nanocomposite coatings can be used safely^20,21^. The development of Poly(urethane-esteramide)/TiO_2_ nanocomposites based on castor oil as anticorrosive and antimicrobial coatings was studied, and the combination of ester and urethane moieties in the polymer chain improved its physico-mechanical and chemical resistance properties^22^. Protective paints based on PEA resins as a binder was described and assessed for use in protective coating compositions. These prepared PEAs were made by partially substituting phthalic anhydride with pyromellitimide acetic acid as a novel source of dibasic acid^23^. The synthesized alkyd/spherical ZnO nanocomposites based on Sunflower oil (SFO) were studied. By analyzing improvements in the mechanical, thermal, physical, and anticorrosive properties of the resins, the significance of nano-filler dispersion was made clear^24^. An exceptional anticorrosive coating made of nano iron oxide impregnated in alkyd (NIAC) is prepared and characterized. The effectiveness of NIAC to suppress corrosion was investigated using the weight-loss technique^25^. S.F. fatty acid has been used to synthesize three various alkyd resins in different ratios with phthalic and maleic anhydride. Sunflower acid oil was evaluated for a wide range of physico-chemical properties, including its fatty acid content, volatile matter, saponification value, iodine value, acid value, viscosity, and other properties^26^. An alcoholysis-polyesterification method was applied to produce an alkyd resin based on palm oil (PO) and strontium oxide/hydroxide nanoparticles. As-prepared SrO/Sr (OH)_2_ nanoparticles were evenly dispersed in the reaction solutions, forming a stable suspension that effectively increased the reaction rate for both alcoholysis and polyesterification in comparison to NaOH^27^. Some chemical, thermal, and abrasion resistance properties of nanocomposite alkyd resin based on inorganic nanoparticles were investigated. To produce nanocomposite coatings, SiO_2_ and TiO_2_ nanoparticles of various compositions were chosen and mixed with alkyd resin^28^. The mechanical characteristics of an alkyd binder enhanced with surface-modified colloidal nano silica were examined and reported^29^. In this research work, we aimed to prepare new modified alkyd and PEA as nanocomposite binders that are based on bio ZnO and bio CuO/ZnO nanoparticles. The newly developed materials were characterized for their corrosion resistance applications, physico-mechanical characteristics as well as chemical resistance properties, and the obtained results demonstrated various novel physical and mechanical properties in addition to promising corrosion-resistance properties for steel substrates exposed to a saline corrosive medium.

## Experimental

### Materials and methods

All the chemicals and solvents were either obtained locally or from global firms. Phthalic anhydride (PA) and diethanolamine (DEA) were purchased from Sdfine Indian. Salicylic acid was a product of Alpha Chemika Co., India. Sunflower oil fatty acid (SOFA) was obtained from Eagle Company, Egypt. Xylene and mineral turpentine products were obtained from Misr Petroleum Company, Egypt. Glycerol (Glys) and phenolphthalein were purchased from the El-goumhouria Co., Egypt. Potassium hydroxide was brought from DOP ORG.KIMYA, Turkey. Zinc acetate dehydrates was acquired from Acros Company (Belgium).

### Synthesis of salicylic diethanolamine (SDEA) (modifier I)

**Figure 1:**
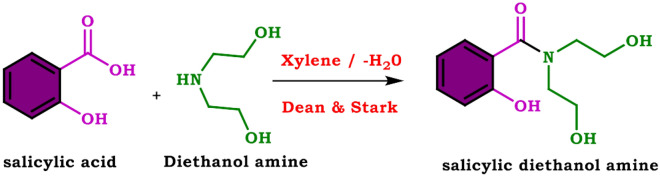
Preparation of salicylic diethanolamine (SDEA).

Diethanolamine (0.5 mol, 52.5 mL) and salicylic acid (0.5 mol, 69 g), were dissolved in 10 mL of xylene and placed in a round-bottomed flask fitted with Dean & Stark. The mixture was allowed to reflux on a hot plate until a (0.5 mol (9 mL) of water was collected (around 6 h). The product was allowed to cool to room temperature. The excess of xylene was removed by using a rotatory evaporator, to obtain a clear pale brown viscous material. The aforementioned steps are illustrated in Fig. 1.

#### Preparation of SDEA-modified PEA

**Figure 2:**
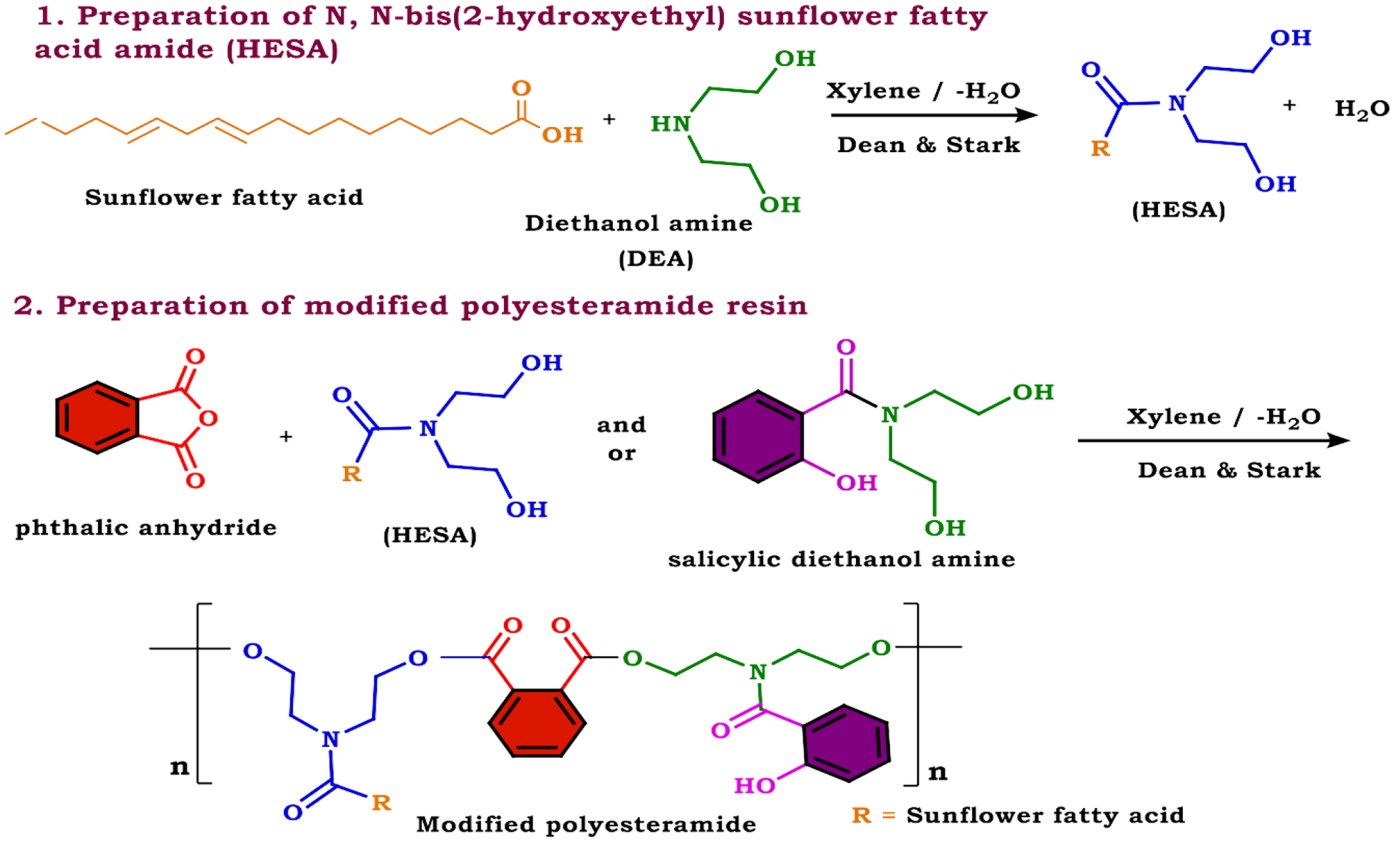
Preparation of SDEA-modified PEA.

A modified PEA resin was produced according to the previously reported protocol^24,30^. The preparation involved two steps. Step 1: For the preparation of monoglyceride (HESA), DEA (0.5 mol, 52.5 g), Sunflower oil fatty acid (0.5 mol, 140 g), and xylene (50 mL) were mixed in a round-bottomed flask fitted with Dean & Stark to remove the all produced water. Step 2: the preparation of SDEA-modified PEA resin was carried out as shown in Fig. 2, by partial replacement of Glycerol (Glyc) with SDEA as the source of the polyol, while Phthalic anhydride (PA) is the source of the polybasic acid, in the presence of 10% xylene by volume. The whole ingredients were placed in a 250 mL round-bottomed flask fitted with Dean & Stark. It should be emphasized that for each set of formulations, the total number of hydroxyl and acid equivalents for different runs was kept constant, and the partial replacement of glycerol with SDEA was done. Additionally, the computation of the water freed can be a helpful tool for monitoring the esterification process to establish the theoretical yield reflected in Tables 1 and 2. Cobalt, zirconium, and calcium octoates were added at concentrations of 0.04, 1.0, and 0.05% based on the metal/solid resin, respectively.

**Table 1:** Resin constants of (SDEA)-modified polyesteramide resins.

Resin no.	Ingredients	E_o_	E	e_A_	e_B_	F	m_o_	R	K	W_gm_	H_2_O Off mL
II_a_	HESA (1.00)	0.260	184	–	0.260	2	0.130	1.00	1.00	47.73	2.33
	SDEA (0.00)	–	–	–	–	–	–			–	
	PA	0.259	74.1	0.259	–	2	0.130			19.19	
				0.259	0.260		0.260			66.92	
II_b_	HESA (0.90)	0.234	184	–	0.234	2	0.117	1.00	1.00	43.10	2.33
	SDEA (0.10)	0.026	113	–	0.026	2	0.013			2.94	
	PA	0.259	74.1	0.259	–	2	0.130			19.19	
				0.259	0.260		0.260			65.23	
II_c_	HESA (0.80)	0.208	184	–	0.208	2	0.104	1.00	1.00	38.3	2.33
	SDEA (0.20)	0.052	113	–	0.052	2	0.026			5.87	
	PA	0.259	74.1	0.259	–	2	0.130			19.19	
				0.259	0.260		0.260			63.36	
II_d_	HESA (0.70)	0.182	184	–	0.182	2	0.091	1.00	1.00	33.48	2.33
	SDEA (0.30)	0.078	113	–	0.078	2	0.039			8.81	
	PA	0.259	74.1	0.259	–	2	0.13			19.19	
				0.259	0.260		0.260			61.48	

HESA, hydroxy ethyl sunflower amide; PA, phthalic anhydride; SDEA, salicylic diethanol amine; E, equivalent weight; e_A_, number of acid equivalent; e_B_, number of hydroxyl equivalent; e_0_, total equivalent present at the start of the reaction; F, functionality; K, alkyl constant (m_0_/e_A_); R, ratio of total-OH groups to total-COOH groups (e_B_/e_A_).

**Table 2:** Resin constants of (SDEA)-modified alkyd resins.

Resin no.	Ingredients	e_o_	E	e_A_	e_B_	F	W_mg_	m_o_	R	K	H_2_O Off (mL)
I_a_	G (1.00)	0.260	30.7	–	0.260	3	7.98	0.086			
	SDEA (0.00)	–	–	–	–	–	–	–			
	LOFA	0.111	280	0.111	–	1	31.08	0.111	1	1.04	2
	PA	0.149	74.1	0.149	–	2	11.04	0.074			1.34
				0.260	0.260		50.1	0.271			3.34
I_b_	G (0.90)	0.234	30.7	–	0.234	3	7.18	0.078			
	SDEA (0.10)	0.026	113	–	0.026	2	2.94	0.013			
	LOFA	0.111	280	0.111	–	1	31.08	0.111	1	1.06	2
	PA	0.149	74.1	0.149	–	2	11.04	0.074			1.34
				0.260	0.260		52.01	0.276			3.34
I_c_	G (0.80)	0.208	30.7	0.000	0.208	3	6.39	0.069			
	SDEA (0.20)	0.052	113	0.000	0.052	2	5.87	0.026			
	LOFA	0.111	280	0.111	0.000	1	31.08	0.111	1	1.07	2
	PA	0.149	74.1	0.149	0.000	2	11.04	0.740			1.34
				0.260	0.260		53.94	0.280			3.34
I_d_	G (0.70)	0.182	30.7	0.000	0.182	3	5.59	0.060			
	SDEA (0.30)	0.078	113	0.000	0.078	2	8.81	0.039			
	LOFA	0.111	280	0.111	0.000	1	31.08	0.111	1	1.09	2
	PA	0.149	74.1	0.149	0.000	2	11.04	0.074			1.34
				0.260	0.260		55.86	0.264			3.34

LOFA, long oil fatty acid; PA, phthalic anhydride; SDEA, salicylic diethanol amine; E, equivalent weight; e_A_, number of acid equivalent; e_B_, number of hydroxyl equivalent; e_0_, total equivalent present at the start of the reaction; F, functionality; K, alkyl constant (m_0_/e_A_); R, ratio of total-OH groups to total-COOH groups (e_B_/e_A_).

IR (KBr): ν_max_ (cm^−1^) = 3348 (OH), 2951 (aliph. CH), 1628 (CON), 1585 aromatic C=C stretching frequency, 760 (Ring stretching vibration of aromatic nuclei).

^1^HNMR (δ, ppm) = 3.491 the presence of CH_2_ peaks attached to free hydroxyl, with amide nitrogen and amide carbonyl peaks present at 3.491 ppm, 3.56–3.745 ppm and 2.6–3.0 ppm respectively, 5.5 existence of OH peaks, 7.0–8.0 peaks present in the aromatic region ^31^**.**

#### Preparation of alkyd resins via a solvent process

**Figure 3:**
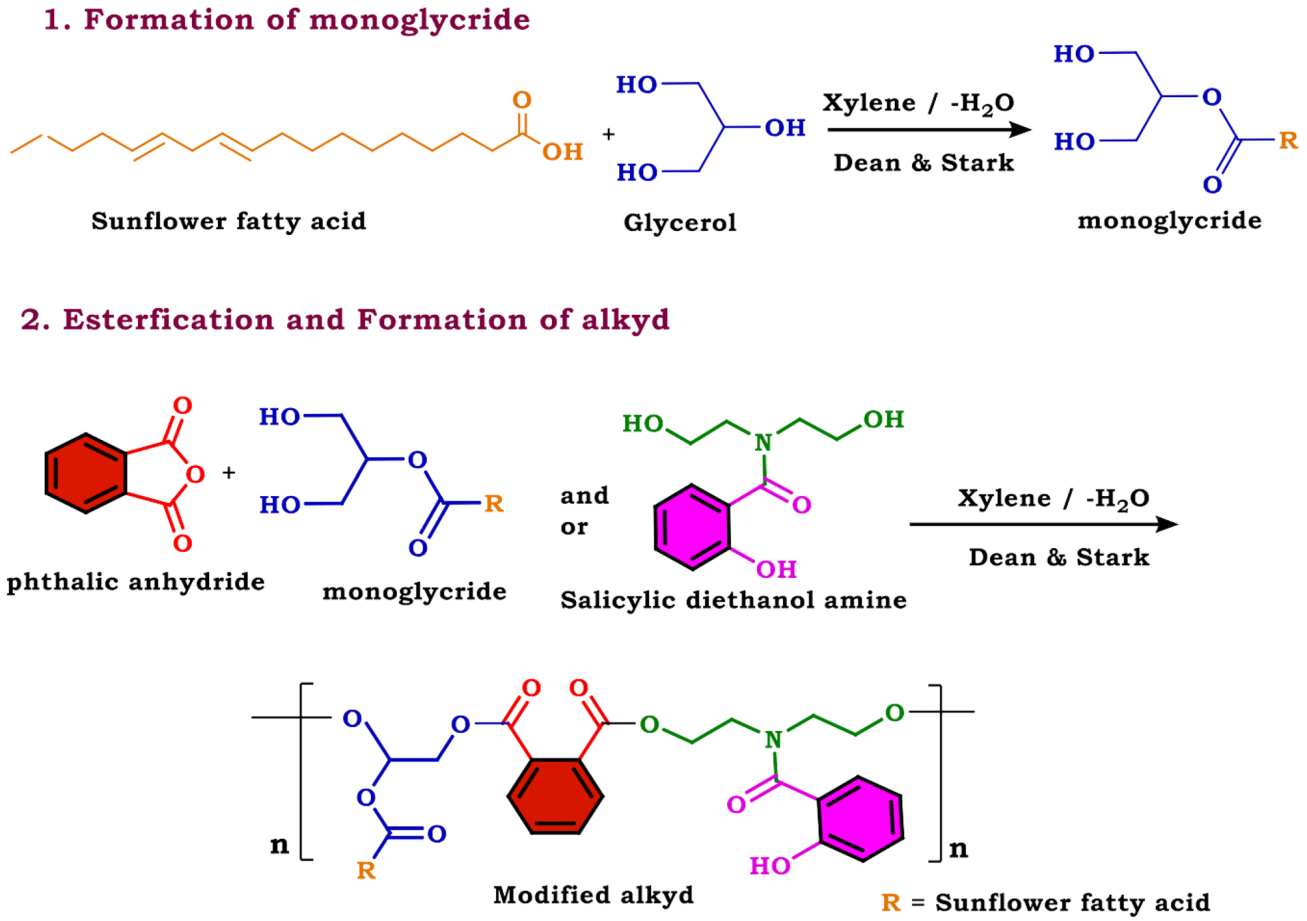
Preparation of modified alkyd resin.

A modified alkyd resin was produced according to the previously reported protocol^26,27,32–38^**.** According to Fig. 3, different modified alkyd resins were prepared by partial replacement of Glycerol (Glyc) with salicylic diethanolamine (SDE) as the source of the polyol's ingredients, whereas the polybasic acid is originated from the phthalic anhydride (PA). All ingredients were added to a 250 mL round-bottomed flask equipped with Dean & Stark apparatus in the presence of 10% xylene (wt/v). It should be emphasized that each set of formulations kept the overall amount of acid and hydroxyl equivalents for different runs constant and replaced some of the glycerol with SDEA. Additionally, the computation of the water freed can be a helpful tool for monitoring the esterification process to establish the theoretical yield reflected in Tables 1 and 2. The temperature was raised up to 240–250 °C and maintained at this range. The reaction was monitored by periodic determination of acid value (AV) of the mixture to the desired number (10–14) mg KOH/g of the resin. Cobalt, zirconium, and calcium Octoates were added at concentrations of 0.04, 1.0, and 0.05% based on the metal/solid resin, respectively.

#### Green synthesis of bio-zinc oxide nanoparticle

Pomegranate peels (POP) were extracted from the local market in Egypt in a high-speed blender (800ES blender, 230 V, 50 HZ, 330 Watt. 1 model BB90E, USA). The fresh POP was blended with distilled water for two hours, filtered, and collected to obtain the extract^39^.

Zinc nitrate hexahydrate and an extract aqueous of Pomegranate peels were used in the production of ZnO NPs according to A.S. Shaban et al.^40^.

#### Green synthesis of CuO/ZnO50/50 nanoparticles

**Figure 4:**
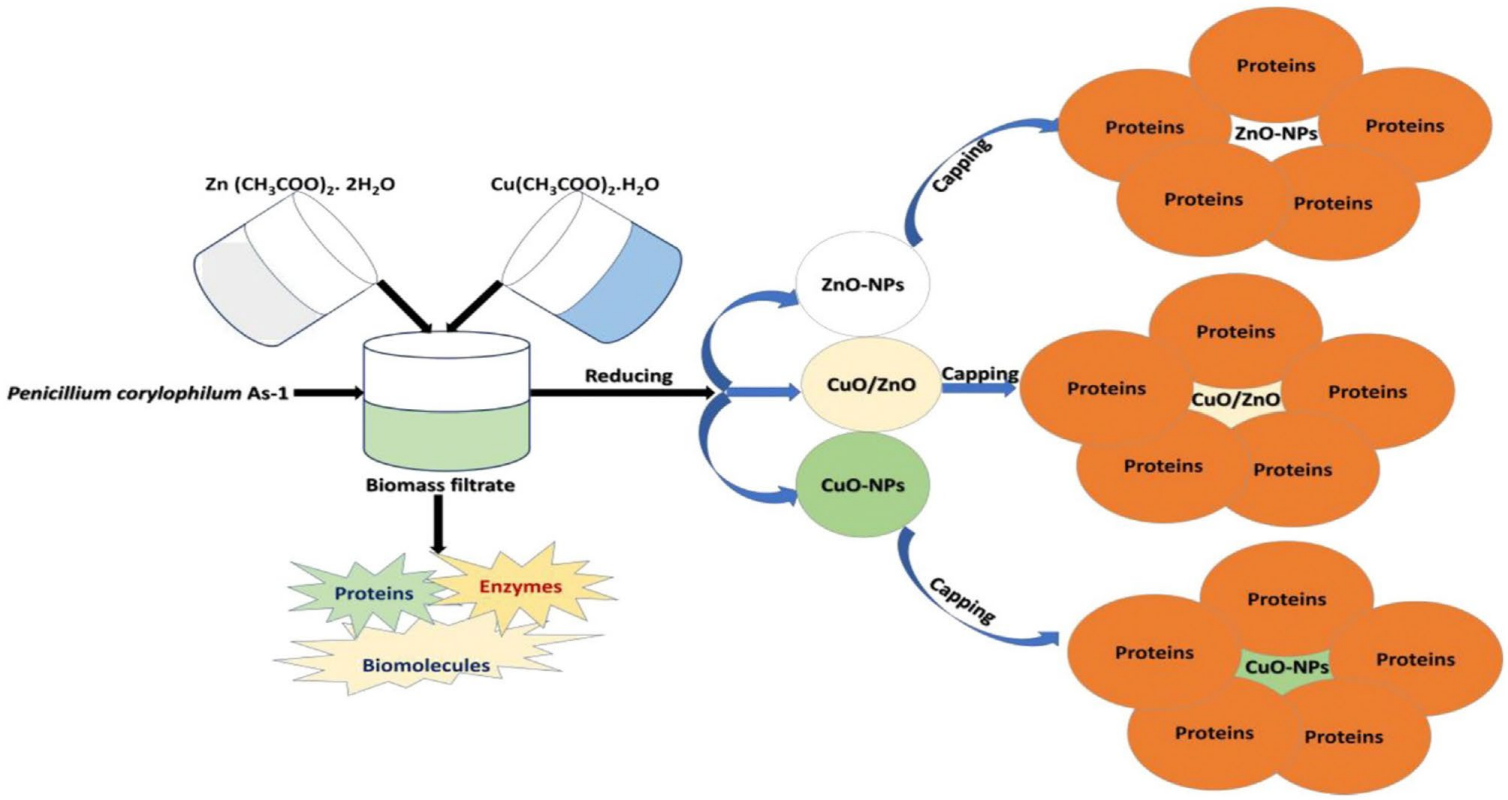
Biosynthesis of CuO / ZnO NPs using biomass filtrate of *P. corylophilum* As-1^41^.

For the preparation of CuO/ZnO50/50, 0.5 g of Cu(CH_3_COO)_2_.H_2_O and 0.55 g of Zn (CH3COO)_2_.2H_2_O were dissolved in 20 mL of dis. H_2_O and added dropwise to 80 mL biomass filtrate to get a final concentration of 5 mM for composite. The composite solution's PH was set at 10 by NaOH (5 M) which was added dropwise under stirring. The formed precipitates were collected and washed twice with dis. H_2_O and oven-dried at 80 °C for 48 h as shown in Fig. 4^41^**.**

#### Preparation of bio CuO/ZnO nanocomposite modified PEA, and bio ZnO nanocomposite modified Alkyd and their incorporation with a high gloss paint formulation

The prepared modified polyesteramide resin and alkyd resin (80 wt% solid content) were mixed individually with 10 wt% xylene and turpentine (1:1) mixing ratio.

**Figure 5:**
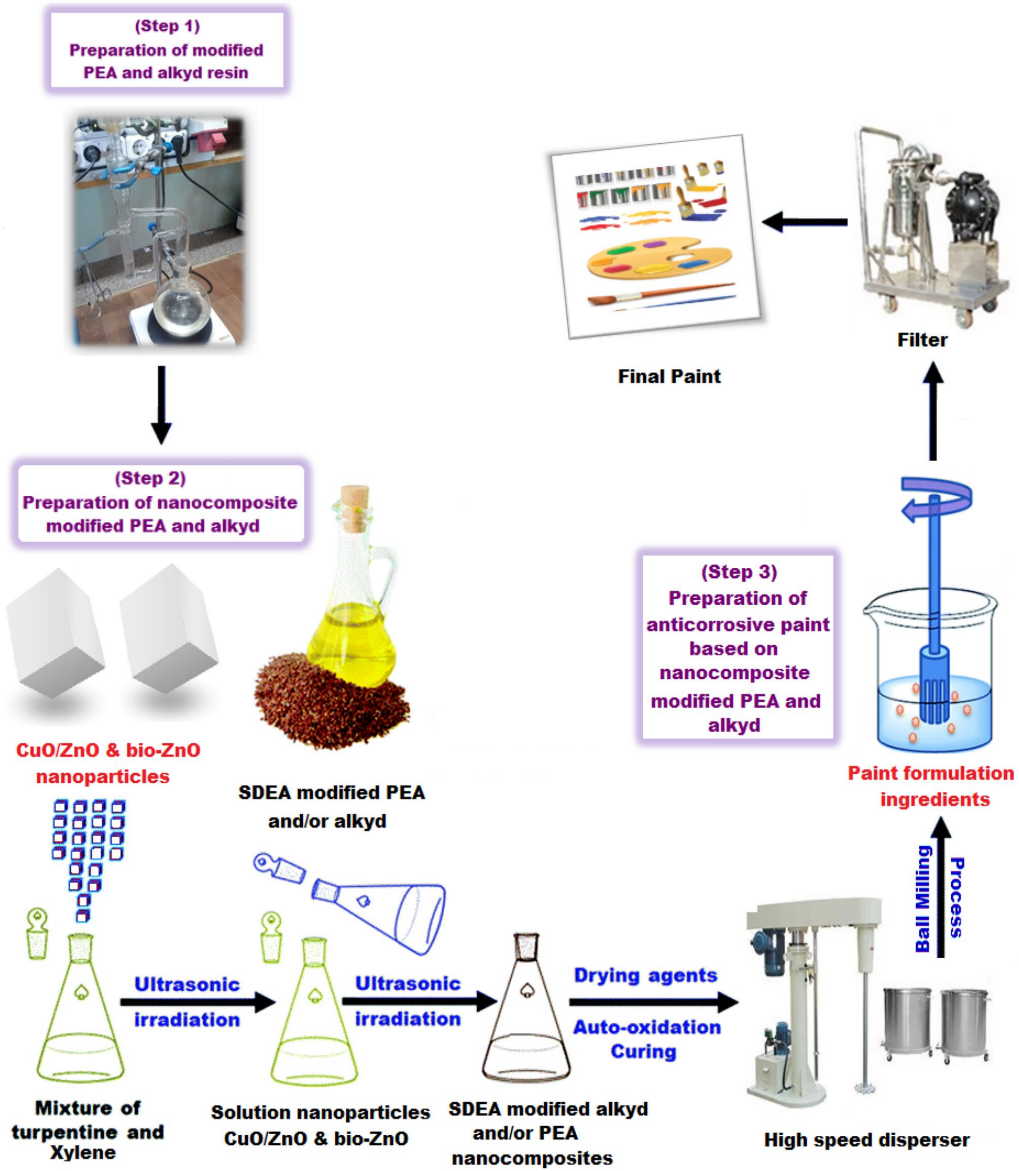
Preparation of nanocomposite modified PEA and alkyd resin.

The desired amount of driers, including Co, Mn, and Ca octoate as a mixed driers solutions, were added by weight of the total formulation, respectively. These driers were mixed under constant stirring to produce homogeneous solutions. Two types of nanoparticles, bio CuO/ZnO and bio ZnO have been selected and used in a concentration of 0.1 wt% to prepare the nanocomposite-modified PEA and alkyd, the prepared nanocomposites were cured using the solution casting technique. CuO/ZnO, ZnO nanospheres of a concentration of 0.1% were sonicated for 15 min. in a mixture of turpentine and methylbenzene (1:1 by volume). The modified PEA and/or modified alkyd resin (80 wt% solid content to (0.1%) wt% CuO/ZnO and/or bio ZnO nanosphere solution) was solubilized in 10 wt% of the utilized solvent under continuous stirring^42^**.** The process is simplified in Fig. 5. After the preparation of nanocomposite resins, the prepared nanocomposite modified resins were added to the paint formulation to create the high gloss coating compositions. Table 3 lists the ingredients in the produced paint formulation either based nanocomposite modified PEA and/or based on bio ZnO nanocomposite modified alkyd resins. The steps of the process have been presented in Table 3, and presented in Fig. 5. The prepared coating formulation’ compositions were applied to steel panels using a brush. Every effort was made to keep the dry film thickness at around 120 + or − 5 µm^27,35–37,43,44,45^.

**Table 3:** Coating formulation of high gloss paint and preparation steps.

Prepared nanocomposite alkyd/or nanocomposite polyester amide resin	50
Xylene	5
Stirring 3 min. for good mixing	
Anti-settling gel	3
Stirring for 5 min. for completely miscible	
F umed silica	0.5
Stirring for 5 min. for completely soluble	
Anti-terra U	1
Titanium dioxide	20
Talk powder	15
Xylene	7
CaCO_3_	6
High speed until reach to optimum fineness (be careful, Temperature should be up to 45 °C	
Low speed for the following	
Prepared nanocomposite alkyd/or nanocomposite polyester amide resin	30
Dryers	0.5
Xylene	4
Iso-butanol	2.5
Methyl ethyl ketoxime	03
UV. stabilizer	0.1

### Characterization

#### Bruker FT-IR analyzer

IR Spectra were determined using KBr-disk technique on a Shimadzu 440 spectrophotometer within the range of 400–4000 cm^−1^.

#### ^1^HNMR spectra (DMSO-d6)

^1^HNMR spectra (DMSO-d6) were obtained on a JOEL spectrometer 500/125 MHz, chemical shifts were measured in *δ *ppm, relative to TMS as an internal standard (= 0 ppm).

#### Degree of polymerization (DP)

The acid value, (an industrial measurement for the conversion that can be obtained by titrating with potassium hydroxide solution in ethanol), was served as a representation of the DP of the resins (KOH) ASTM D1980-87(1998)^46^.

#### Thermal analysis

Thermogravimetry analysis of the coated sample was carried out using a TGA instrument (STA model 650) with a heating rate of 20 °C/min in a 45–950 °C temperature range.

#### Scanning electron microscopy (SEM)

Surface morphologies of the prepared nanocomposite and coated samples were observed with a scanning electron microscope (SEM, Joel Jsm 6360LA-Japan).

#### Transmission electron microscopy (TEM)

Samples were observed under transmission electron microscopy (TEM) on JEOL (GEM-1010) at an operating voltage of 76 kV.

#### Method of testing and evaluation

All resins were adjusted to a solid content = 60% (according to ASTM Method, D1644-01, (Reapproved 2012), and the performance film properties were examined using the following test methods: the preparation of the steel panels D609-17; measurement of film thickness according to ASTM method D1005-13. The specular gloss measurements were performed according to ASTM method D523-18, and film hardness was measured using a pencil hardness tester according to ASTM method D3363-11. Finally, adhesion was tested using a cross-hatch cutter, according to ASTM method D3359-17, and flexibility was determined as per the ASTM method D522-17.

#### Anticorrosive studies

The salt spray test was used to determine the corrosion resistance of the painted panels. The guidelines and test procedures are described in the international standard ASTM B117.39 The salt fog solution consists of sodium chloride (5%wt). The nano-coated surfaces were exposed to a C&W salt fog cabinet (model SF/MP/AB100, United Kingdom) fitted with a CAC 200/310 concord compressor station (Japan). The salt spray cycle was subdivided as follows: (1) exposure of the tailored nano-coating samples to salt fog at 8 mL min^−1^ (sprinkled by a nozzle for 30 min); and (2) a constant cabinet temperature of 35 °C without salt injection (15 min). The electrolyte was replaced with a fresh solution after 1000 h of exposure. Water repellency is a major factor that contributes to corrosion protection because it improves the anti-stick characteristics of tailored nano-surfaces toward aqueous particles, leading to high anticorrosion performance^24,47^.

## Results and discussion

### Spectral data characterization

#### FT-IR and ^1^HNMR of SDEA modified (PEA)

SDEA-modified PEA resin was confirmed by the FT-IR and ^1^HNMR analyses as illustrated in S (3) and S (4), respectively.

IR (KBr): ν_max_ (cm^−1^) = 3449 (OH), 3008 (arom. CH), 2926 (aliph. CH), 1728 (COO stretching of ester linkages), 1632 (CON amide carbonyl), 746 (Ring stretching vibration of aromatic nuclei).

^1^HNMR (δ, ppm) = Characteristic peaks for chain –CH_2_– appears at δ = 1.14–1.99 and terminal –CH_3_ appears at δ = 0.84 ppm. The peaks of CH_2_ attached to amide carbonyl (> N–(C=O)–CH_2_) at δ 4.5 ppm, and amide nitrogen ((–CH_2_)_2_ N–C=O) at δ 3.5 ppm. The peaks for protons of the –CH=CH moiety appear at δ 5.29–5.31 ppm. The proton of the phthalic moiety's aromatic ring and the proton of the xylene moiety's aromatic ring at a concentration between 6.9 and 7.9 ppm^31^**.**

#### Spectral analysis of SDEA-modified alkyd resin

Alkyd resin's structural characteristics were investigated using FT-IR shown in S (5) and ^1^HNMR in S (6).

IR (KBr): ν_max_ (cm^−1^) = 3446 (OH), 3008(arom. CH), 2927 ((C–H) CH_2_ asymmetric stretching), 2855 ((C–H) CH2 symmetric stretching), 1738(COO stretching of ester linkages), 3009(arom. CH), 1599 (stretching frequency of alkene and aromatic band), 746 (Ring stretching vibration of aromatic nuclei).

^1^HNMR (δ, ppm) = Characteristic peaks at δ = 0.8–0.9 due to terminal –CH_3_ group, peaks at δ = 1.15–154 ppm. Due to the protons of CH_2_ groups attached next to the above terminal methyl group. Peaks at δ = 2.00–2.76 ppm for CH_2_ of double allylic protons. Characteristic peaks at δ = 4.14–4.39 ppm is for protons of the glyceride moiety, and the peak at δ = 5.33 ppm is due to existence of OH peaks, the peaks for protons of the –CH=CH moiety appear at δ = 6.75–6.99 ppm, at δ = 7.125–7.85 ppm are observed for aromatic protons of PA containing resins.

### Confirmation of bio-ZnO nanoparticle by FT-IR analysis and transmission electron microscopy (TEM)

#### FT-IR analysis

S (7) shows the ZnO NPs FT-IR spectrum. The sample has absorption peaks in the range of 800–1600 cm^−1^, which are connected to the presence of organic chemicals. The amide group's C=O stretching vibration band and the existence of COOH and OH groups are shown by the peaks' absorption at 1630 and 3437 cm^−1^, respectively^37^**.** The –CH_2_ and C–C bonds in the aromatic ring are responsible for peaks at 2920 and 1411 cm^−1^, respectively. The peak absorption at 1630 cm^−1^ can also result from stretching vibrations of C=O. A peak at 1039 cm^−1^ also suggests the presence of phenol, alcohol, and amines (aliphatic). The phenols in the capping agent link to the surface of ZnO to create ZnO NPs, while the C=O, C–O=C, and C=C groups of heterocyclic compounds may serve as stabilizers^38,39^**.** Because the peaks in the region between 500 and 900 cm^−1^ are ascribed to metal-oxide (M–O) bonds, the FT-IR spectrum then absorbs the peak at 875 cm^−1^, indicating the stretching vibrations of ZnO^40^.

#### Morphological analysis

**Figure 6:**
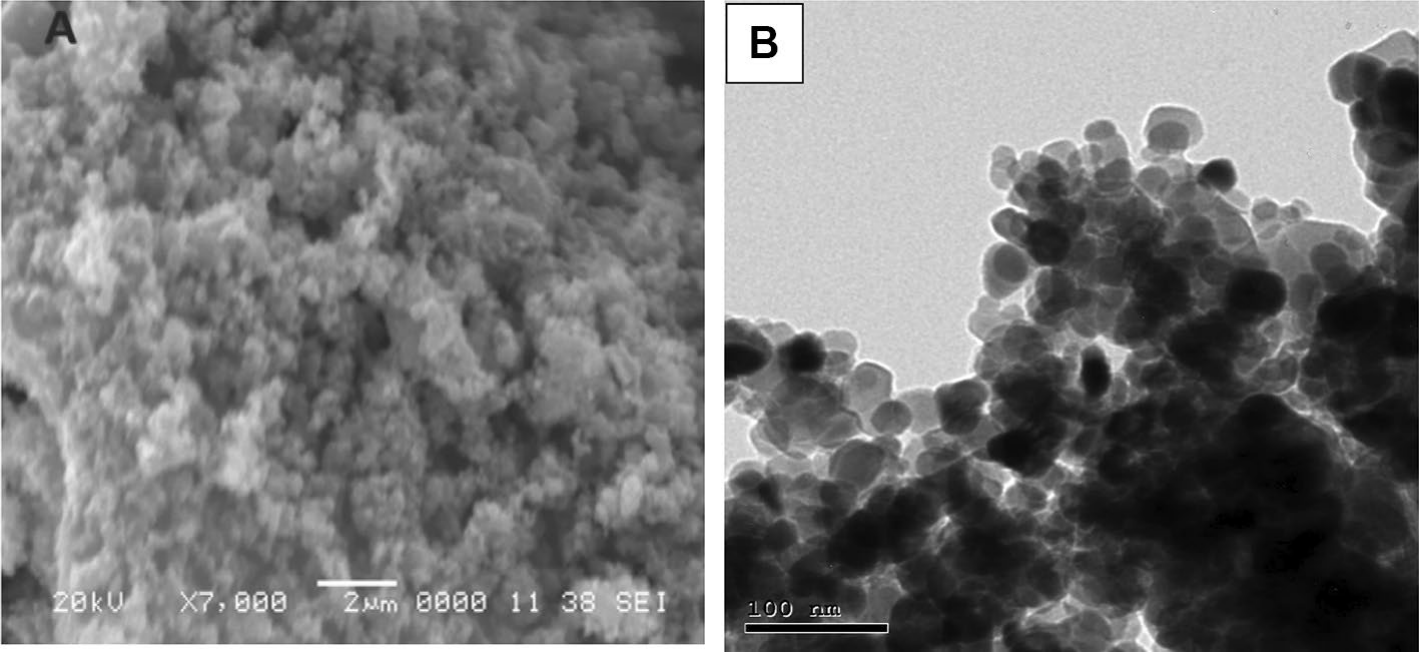
Characterization of the biosynthesized ZnO NPs (**A**) denotes SEM image, (**B**) denotes TEM image.

To study the morphology of ZnO NPs, SEM measurements were carried out. The SEM micrographs depicted in Fig. 6A show how the obtained ZnO NPs are approximately spherical in form with aggregation as a result of their interaction with water and the intramolecular Van-der Waals, magnetic, and electrostatic interactions. TEM proved the homogeneity and nanoscale of ZnO NPs. The form the nanoparticles generated is of a spherical type with an average size found to be 20–40 nm as indicated in Fig. 6B.

### Confirmation of CuO/ZnO nanoparticle by FT-IR analysis and transmission electron microscopy (TEM)

#### (FT-IR) spectroscopy

S (8), shows the FT-IR spectra of the fabricated nano-composites in the range of 400–4000 cm^−1^^31,48–50^. The nanocomposite FT-IR spectra display a variety of clear peaks. The effective synthesis of CuO and ZnO in all the samples might be the cause of the emergence of peaks at low wavelengths from 400 to 700 cm^−1^^51–55^. On the other hand, the observed peak at 1569 cm^−1^ involves the bending vibrations of protein (NH). Bands at 1408 and 1324 cm^−1^ are allocated to CN vibrations of amines (aromatic and aliphatic)^27^, whereas that at 864 cm^**−**1^ may be is ascribed to CH and CC of alkene^56^.

#### Scanning electron microscopy (SEM)

**Figure 7:**
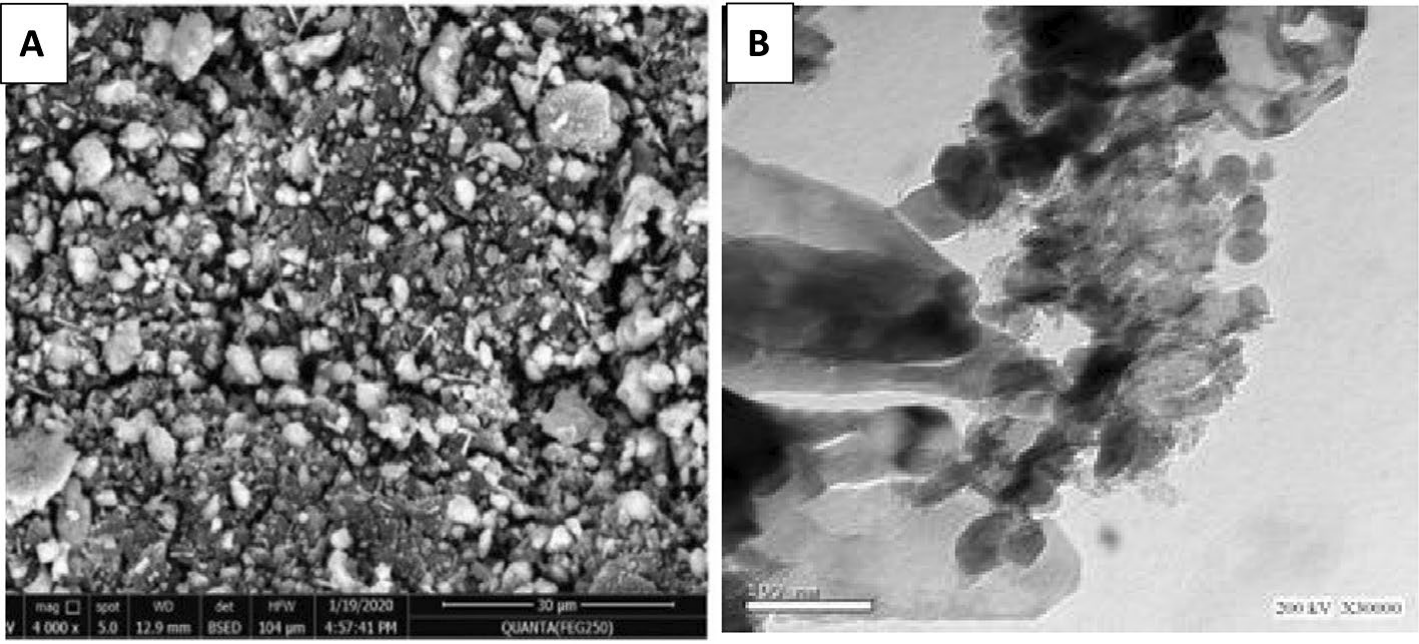
SEM image (**A**) and TEM image (**B**) for CuO/ZnO.

SEM was utilized to analyze the surface morphology of the biosynthesized NPs as seen in Fig. 7A; the result proved almost spherical NPs with few agglomerations. These agglomerations may be linked to NPs' increased propensity for forming huge clusters^57^.

#### Transmission electron microscopy (TEM)

Size and form, among other morphological traits, play significant roles in the actions of NPs. This led to the creation of biosynthesized CuO/ZnO nanocomposites. The efficient biosynthesis of nano-spherical forms in various sizes was confirmed by the TEM pictures, the CuO/ZnO nanocomposite appeared as spherical particles having a size with an average of ~ 30–55 nm in Fig. 7B.

### Characterization of prepared CuO/ZnO nanocomposite-modified PEA and bio-ZnO nanocomposite-modified alkyd resins

#### FT-IR Spectroscopy of bio-ZnO nanocomposite modified alkyd resins

IR spectrum of CuO/ZnO nanocomposite-modified PEA is shown in S (9).

IR (KBr): ν_max_ (cm^−1^) = 3377 (OH), 1726 (COO group stretching of ester linkages), 3009 (arom. CH), 1599C (stretching frequency of alkene and aromatic band), 746 (Ring stretching vibration of aromatic nuclei). 1121 and 1189 (C–O and C–N stretching respectively), 603 (stretching mode of ZnO).

#### Scanning electron microscopy (SEM) of nanocomposite-modified PEA and nanocomposite-modified alkyd

**Figure 8:**
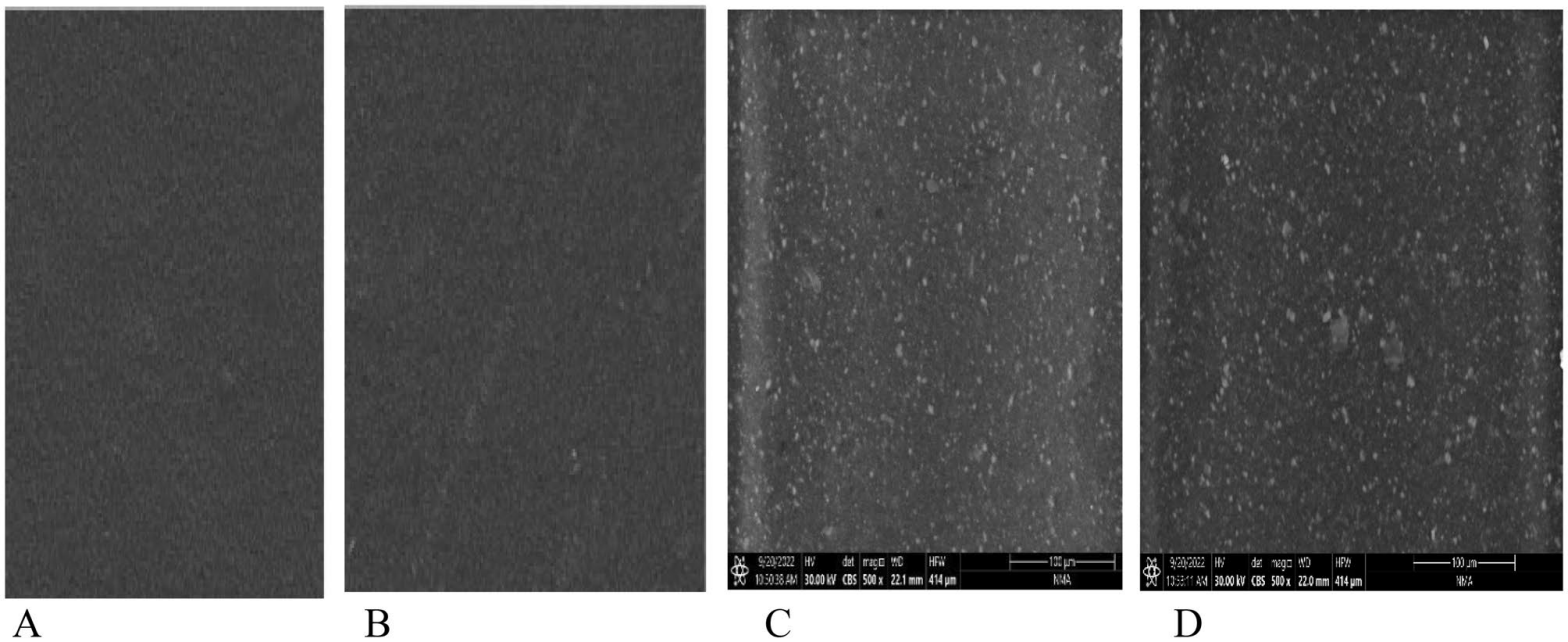
SEM images of blank PEA and alkyd (**A**, **B**), bio CuO/ZnO nanocomposite modified PEA (**C**) and bio ZnO nanocomposite modified alkyd resin (**D**).

SEM images of blank PEA and alkyd resins (A, B), bio CuO/ZnO nanocomposite modified PEA (C), and bio ZnO nanocomposite modified alkyd resin (D). Figure 8 shows SEM images illustrating the states of sample blank PEA and alkyd without modification Fig. 8A and bio CuO/ZnO NPs in the PEA resin Fig. 8B and bio ZnO NPs in the alkyd resin Fig. 8C of to form nanocomposite modified polymers. It’s observed that there’s good roughness for all the samples, either for sample blanks of PEA and alkyd resin or for modified PEA and alkyd nanocomposite. Although the level of roughness was not uniform throughout the sample, it was visible for the good loadings of the proportion of modified nanocomposite PEA and alkyd; also, the images indicated that there’s no agglomeration, and performance and dispersion were good.

### Characterization of prepared paint formulation based on nanocomposite

#### Thermal analysis

##### Thermal analysis of paint based on bio CuO/ZnO nanocomposite modified PEA

**Figure 9:**
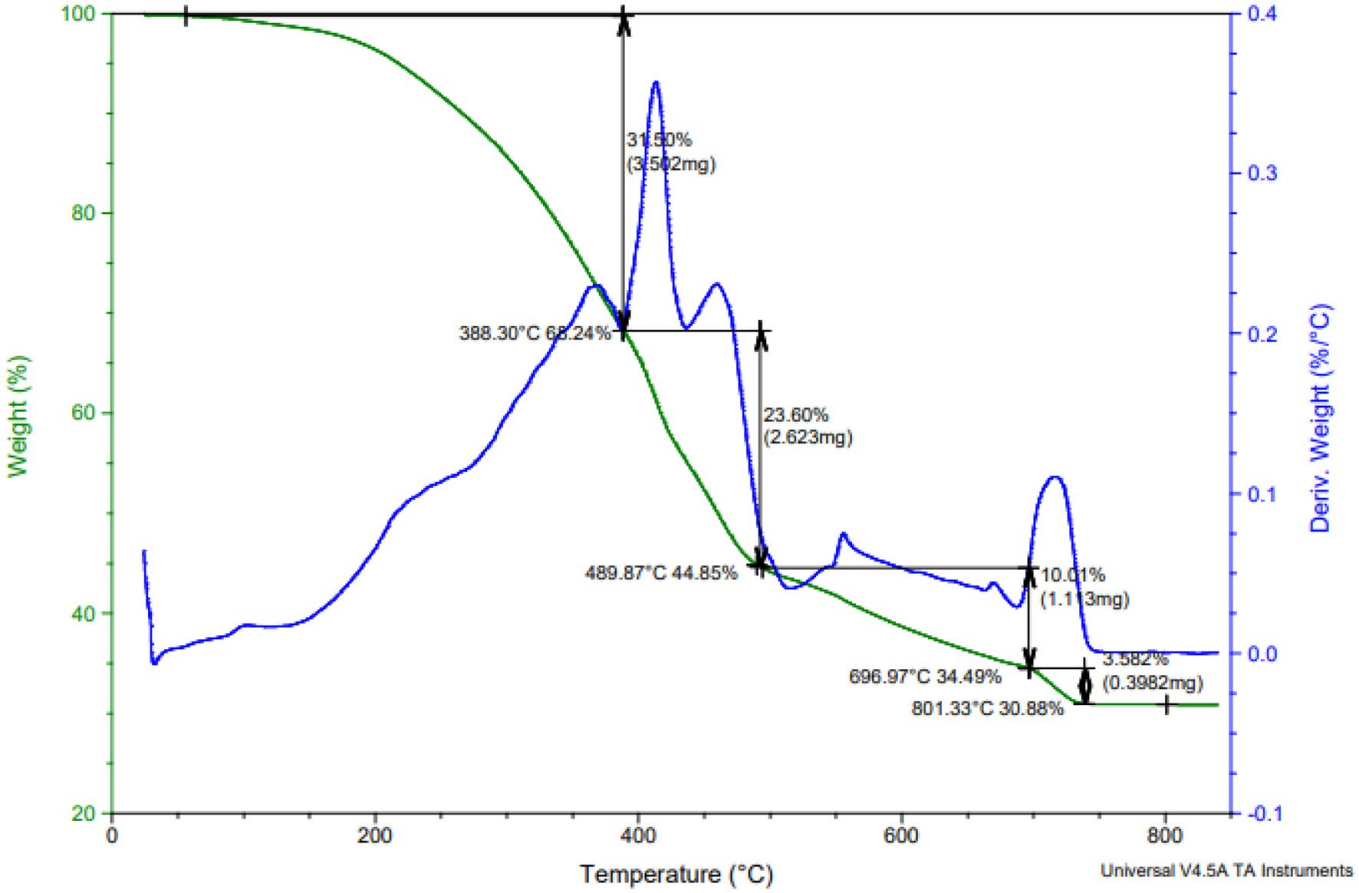
TGA of the dry coated film based on bio- CuO/ZnO nanocomposite modified PEA.

Figure 9 shows TGA of dry coating based on CuO/ZnO nanocomposite-modified PEA sample. The TGA curve of this sample shows four distinct weight loss steps, consistent with the results reported for nanocomposite coating^31^. The coated sample's DTG curve shows four peaks that correlate to different stages of weight loss. The first step in the weight loss in TGA curve and the associated first peak in DTG curve at 388 °C are caused by the decomposition of the aliphatic diol portion (HESA) in the polyesteramide and adsorbed solvent and reacted moieties^31^. The second step of weight loss and the associated second peak at 489.87 °C is caused by the decomposition of ester, ether, and unsaturated bonds and ester amide moieties present in the coating matrix^25,31^. The third weight loss step and the corresponding third peak at 697 °C may be attributed to the decomposition of CuO/ZnO composite modified PEA into the coating matrix^58^. The fourth weight loss step and the related fourth peak at 801 °C may be the cause of the decomposition of some fillers in the coating formulation, such as calcium carbonate, which is converted into calcium oxide (CaO), and also, TiO_2_. Therefore, the high compatibility between the nanocomposite-modified resin and the solid materials in the coating formulation and the good dispersion. So, the obtained results of the thermal gravimetric stability indicated that the final product of the coating based on CuO/ZnO modified PEA has good thermal stability because of the introduced nanocomposite binder, nitrogen elements in the polymer structure, and finally, the high compatibility and high dispersion of the components of the coating formulation.

##### Thermal analysis of paint based on bio ZnO nanocomposite modified alkyd resin

**Figure 10:**
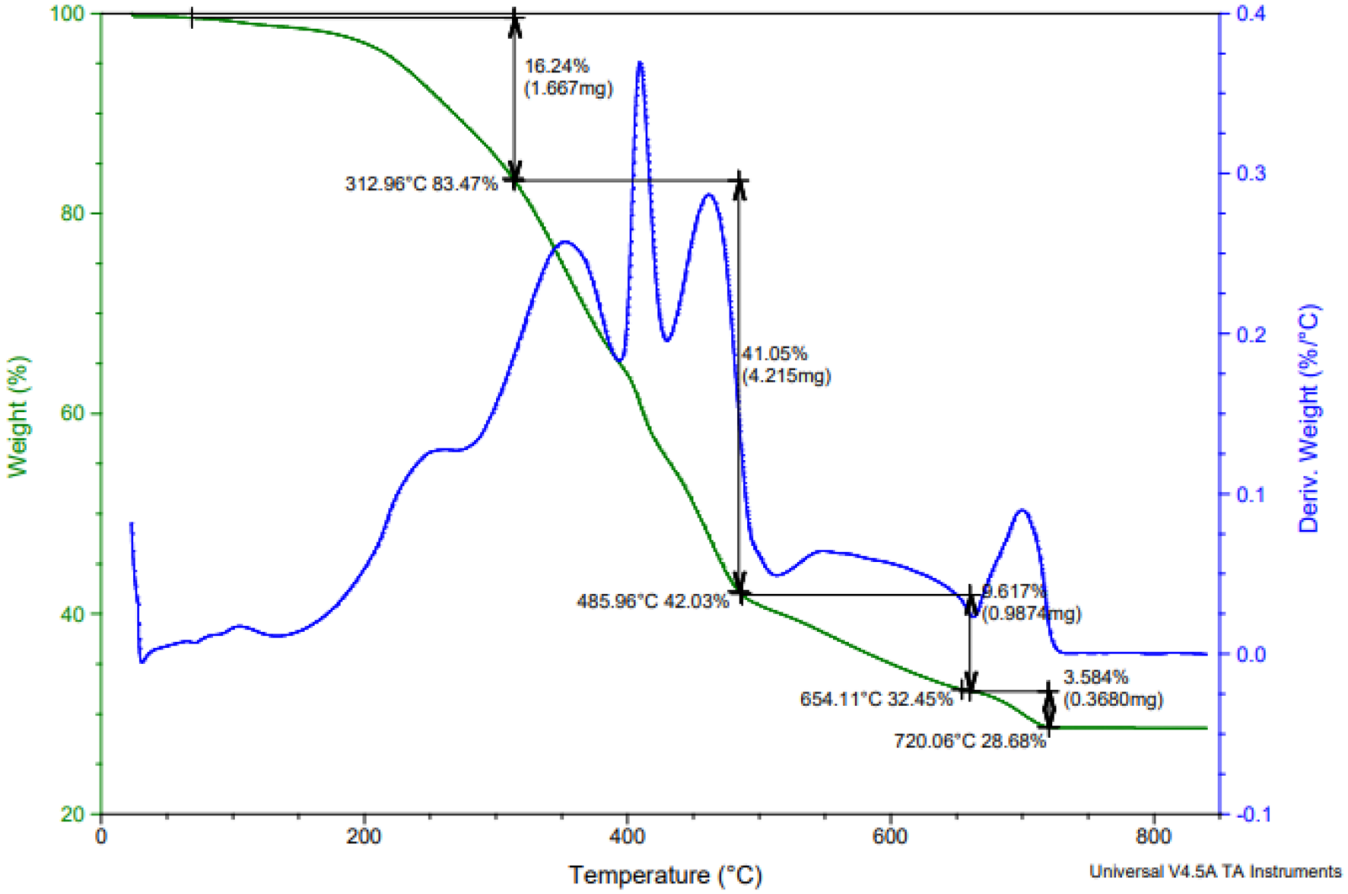
TGA of the coated film based on bio ZnO nanocomposite modified alkyd resin.

Figure 10 shows the similar behaviour of TGA and derivative thermogravimetry analysis (DTG) curves for dry coating based on CuO/ZnO nanocomposite-modified PEA and bio ZnO nanocomposite-modified alkyd samples. But there are a few differences between each of them, because of the presence of nitrogen element which is repeated in the polyesteramide and introduced in CuO/ZnO Np, which combines the advantage of Cu and the advantage of Zn, So, all of these encourage the enhancement of the high thermal stability of these types of coatings. So, we can notice from Figs. 9 and 10 that the obtained results of the dry coating based on CuO/ZnO nanocomposite-modified PEA were more thermally stable than those of the dry coating based on bio ZnO nanocomposite-modified alkyd resin. The coated sample's DTG curve shows four peaks that correlate to different stages of weight reduction. The initial step in the TGA curve's weight loss and the matching first peak in the DTG curve at 31 °C result from the decomposition of the aliphatic diol portion (HESA) in the modified alkyd and adsorbed solvent and reacted moieties^31^. The second stage of weight loss and a subsequent second peak at 486 °C are caused by the decomposition of ester, ether, and unsaturated bonds present in the coating matrix^25,31^**.** The third stage of weight loss and subsequent third peak at 654.11 °C might be attributable to the decomposition of bio ZnO composite modified alkyd resin into the coating matrix^58^. The fourth weight loss step and the fourth peak at 720 °C might be a result of the decomposition of some fillers in the coating formulation, such as calcium carbonate, which is converted into calcium oxide (CaO), and, TiO_2_. Thus, the high compatibility between the nanocomposite-modified alkyd resin and the solid materials in the coating formulation and the good dispersion. So, the obtained results of the thermal gravimetric stability indicated that the final product of the coating based on bio ZnO-modified alkyd is a good thermally stable.

#### (SEM) with (EDX) study of paint-based CuO/ZnO nanocomposite modified PEA and bio ZnO nanocomposite modified alkyd resin

**Figure 11:**
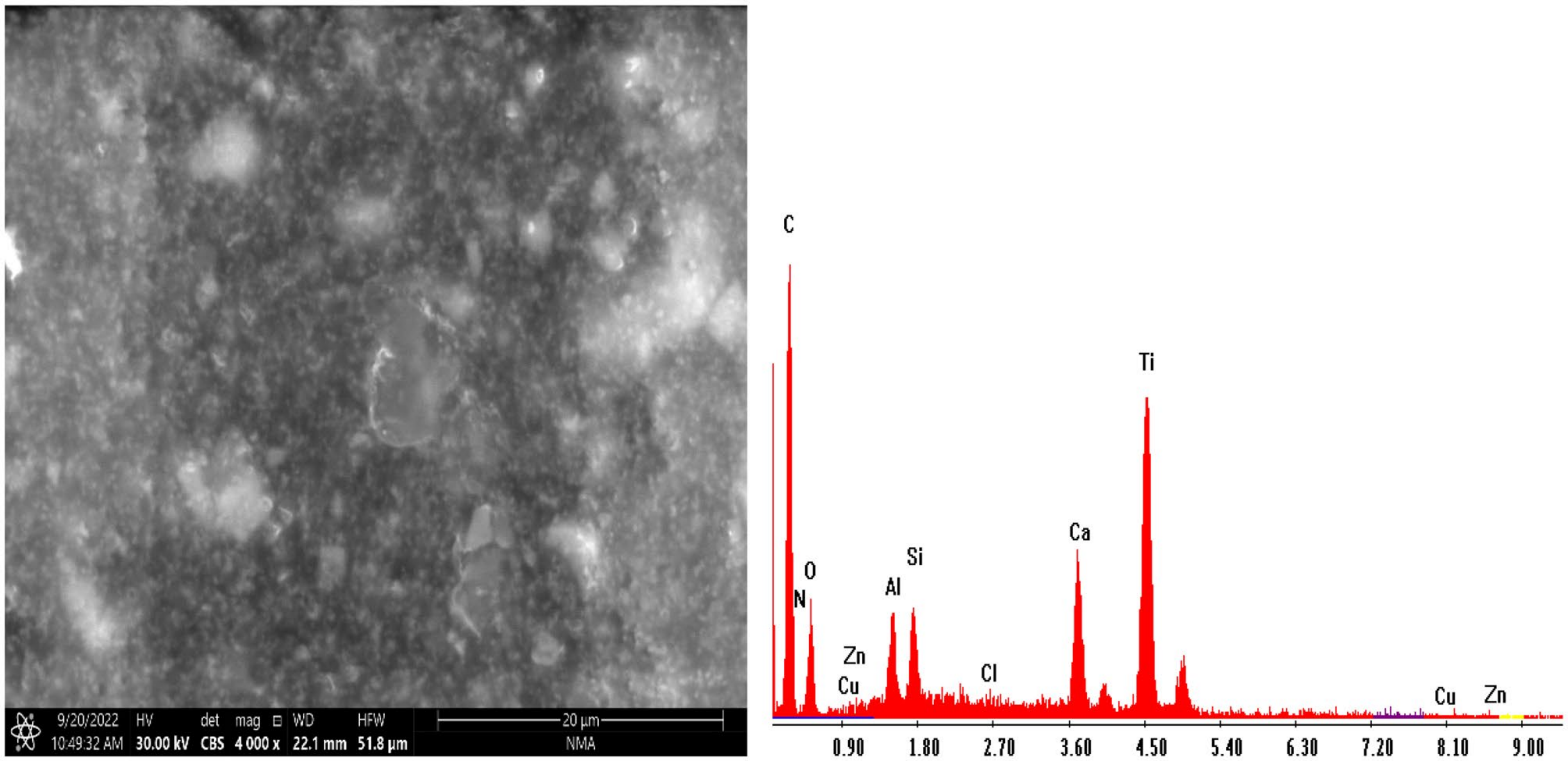
SEM with EDX image for paint-based bio CuO/ZnO nanocomposite modified PEA resin.

**Figure 12:**
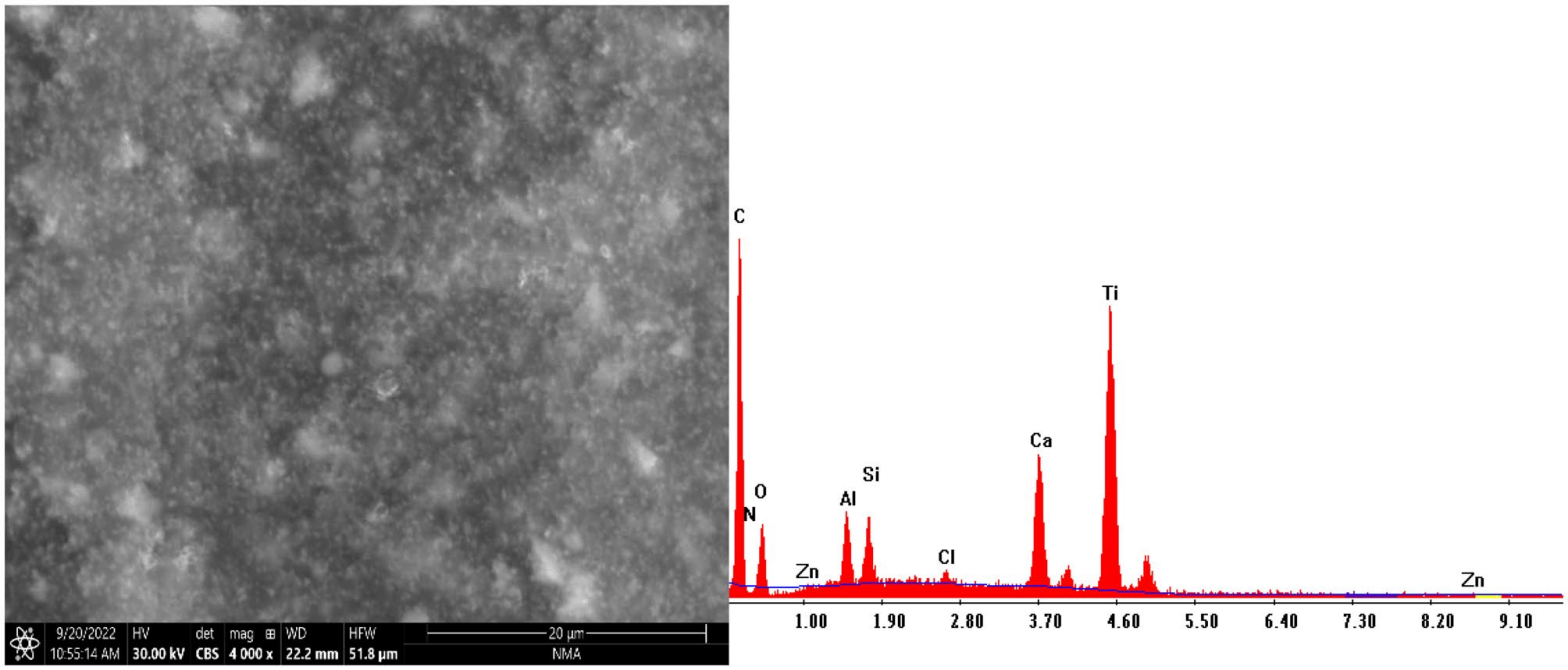
SEM with EDX image for paint-based bio ZnO nanocomposite modified alkyd resin.

SEM micrograph depicted in Figs. 11 and 12 of paint-based CuO/ZnO nanocomposite modified PEA and bio ZnO nanocomposite modified alkyd resin reveals a homogeneous coating without any obvious cracks, which are found to be adequately confined in the modified polymer matrix despite their not well- defined shapes. EDX results suggest that the bio CuO/ZnO and bio ZnO NPs trapped in the polymer matrix. Figures 11 and 12 show bio CuO/ZnO and bio ZnO nanocomposite modified PEA and alkyd confirming that the paint formulation contains bio CuO/ZnO and bio ZnO, carbon, hydrogen, and nitrogen atoms. The peaks observed at 0.3, 0.4, and 0.6 keV correspond to the binding energies of C, N, and O, respectively. These peaks observed at 1.5, and 8.5, belong to Zn, as shown in Fig. 11, while peaks observed at 0.5, 0.8, 8.0, and 8.8 belong to Cu and Zn. Due to the surface plasmon resonance, these optical absorption peaks represent the typical absorption of Cu and Zn nanocrystalline metals.

### Coating characteristics of cured modified resins

#### Mechanical characteristics

We can see from the data shown in Table 4 that the mechanical characteristics of curing panels of modified PEA and alkyd resins were similar in the results that were obtained. The existence of salicylic diethanolamine as a modifier led to significantly improved physio-mechanical characteristics such as scratch hardness, gloss, adhesion, and thermal stability. It was noticed that in the case of coated films based on modified PEA, the mechanical properties were improved more than those based on modified alkyd, and this may be due to the presence of nitrogen element in the PEA. Fatty acids undergo an oxidative cross-linking process, which causes PEA and alkyd coating films to dry. So, as shown in Table 4. The drying time in the stove increases from 2–3 h at 150 °C and this may be owing to the fact that with the increase of the fatty acid content in the resin, the unsaturation part degree was not enough to oxidize, so it required more than 2 h to be hard dry films if they were compared to blank samples of PEA and alkyd resins. The stiff aromatic moiety in the polymer chain is responsible for the increased pencil hardness rating. The hardness value for resins is also increased by the addition of SDEA^35^. The bend test reveals that cross-linked networks have excellent flexibility. Alkyd resins pass the bend test and have strong adhesion qualities because they contain polar ester linkages, either in PEA or in alkyd resins. Table 4 shows that the other important properties like adhesion and gloss of modified PEA and alkyd resins were also enhanced by boosting the replacement in PEA and alkyd. The resin's adhesion was enhanced because of the existence of polar ester bonds. Gloss is enhanced due to the presence of the aromatic PA moiety and the novel modifier (SDEA) in PEA and alkyd resin. All of the resin's gloss properties may be rated as good^24–37,43–45,48,59^.

**Table 4:** Mechanical characteristics of various SDEA- Modified PEA and Alkyd resin.

Sample ID		Film thickness (μ)	Gloss	Scratch Hardness (kg)	Flexibility	Adhesion	Acid value	Colour (Gardner scale)	Specific gravity (28 °C)	Stoving drying time (hr.) at 150 °C
Nanocomposite PEA	II_a_(0%)	40	100	< 1.5	Ex	4B	10	10	0.96	2
	II_b_(10%)	40	104	> 2	Ex	5B	15	15	0.93	2.5
	II_c_(20%)	50	104	> 2	Ex	5B	13	16	0.92	2.5
	II_d_(30%)	50	105	> 2	Ex	5B	12	18	0.92	2.5
Nanocomposite Alkyd	I_a_(0%)	45	100	< 2 kg	Ex	4B	11	10	0.96	2
	I_b_(10%)	50	135	> 2 kg	Ex	5B	12	15	0.93	> 2
	I_c_(20%)	45	124	< 2 kg	Ex	5B	13	16	0.92	> 2
	I_d_(30%)	40	105	< 2 kg	Ex	4B	13	18	0.92	> 2

#### Chemical resistance of dry-coated films

Table 5 lists the resin's film performance in various chemical conditions. All samples of modified and nanocomposite resins had chemical resistance characteristics comparable to the reference sample of PEA, and all resins' alkyd films have excellent water resistance, acid, and solvent resistance except for alkali. The modified resin is fairly resistant to alkali than blank sample, maybe as a result of the PA's abundant stiff aromatic moiety and the content of SDEA in the structure of prepared resins, which increases by increasing the replacement. As a result of the presence of an alkali hydrolyzable ester group, the alkali resistance drops^24–37,43–45,48,59^.

**Table 5:** Chemical resistance of dry coated films based on SDEA- modified nanocomposite PEA and Alkyd resins.

Sample ID	SDEA- modified nanocomposite PEA resins				SDEA- modified nanocomposite Alkyd resins			
	Water resistance	Solvent resistance	Alkali resistance	Acid resistance	Water resistance	Solvent resistance	Alkali resistance	Acid resistance
I_a_,II_a_(0)%	Pass	Pass	Poor	Pass	Pass	Pass	Poor	Poor
I_b_,II_b_(10)	Pass	Pass	Poor	Pass	Pass	Pass	Poor	Pass
I_c_,II_c_(20)	Pass	Pass	Poor	Pass	Pass	Pass	Poor	Pass
I_d_,II_d_(30)	Pass	Pass	Poor	Pass	Pass	Pass	Poor	Pass

The chemical resistances of the unmodified PEA and unmodified alkyd-cured films and nanocomposites were evaluated in comparison to several solutions, including Na_2_CO_3_, H_2_O, and H_2_SO_4_ in Table 5. Applying the coatings to the glass plates allowed for the evaluation of the films' chemical resistance. Plates were then cured for 48 h by being submerged in various solutions and solvents. To stop solvent from penetrating between the plate and the coating, the edges of the plates were coated with paraffin wax. The plates were evaluated and examined for any signs of changes to the coated resins. Comparing the nanocomposite films to unmodified PEA and alkyd films, the nanocomposite films showed greater resistivity and indicated higher resistance in acid solutions. To investigate the coatings' acid stability, an H_2_SO_4_ solution was used. The films demonstrated good resistance to acid. All the films that had been immersed in the H_2_SO_4_ solution afterward showed gloss loss and color changes. The films of unmodified alkyd then blistered, but the films of modified PEA and modified alkyd simply shrank and continued to be acid resistant. Using a 5 wt.% Na_2_CO_3_ solution, the alkali resistance of coatings was evaluated. Within 48 h of soaking, the changes in the coated plates were noticeable. Within two days, all films of unmodified and modified PEA and modified alkyd were removed entirely. The hydrolyzable ester linkages in the PEA and alkyd resin chains were likely the source of the low resistance of alkyd films and their nanocomposites in the alkali media^60,61^**.** Glass plates with coatings that were submerged in distilled water exhibited no change. This is explained by their elevated hydrophobicity. The results of the chemical resistance tests of all samples of coated films made from modified PEA and modified alkyd resin are summarized in Table 5 and are identical.

### Testing corrosion resistance of the coating

**Figure 13:**
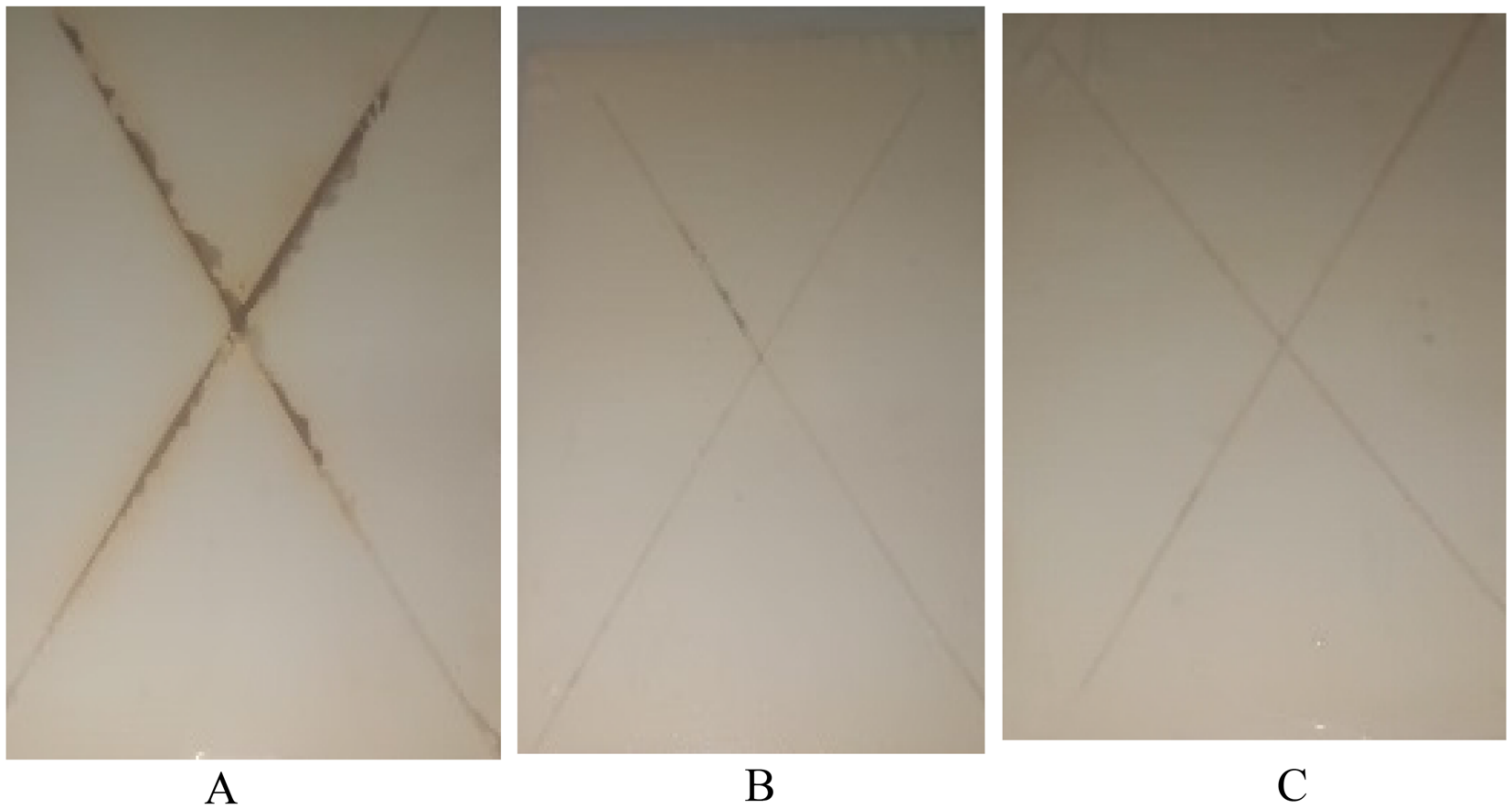
Photographic images (**A**) blank coating, (**B**) coating based on CuO/ZnO nanocomposite modified PEA resin (**C**) coating based on bio ZnO nanocomposite modified alkyd resin.

**Figure 14:**
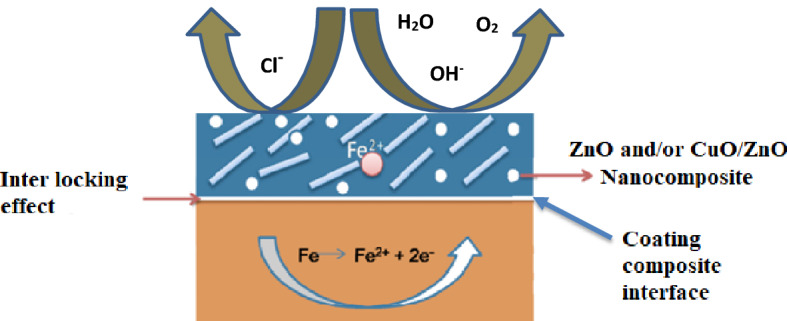
Mechanism of corrosion protection.

Table 6 contains measurements and statistics related to salt spray. Once the films failed to adhere, the salt spray test was discontinued. Figures 13 and 14 illustrate the salt spray test findings with pictures of dried paints made from PEA and alkyd modified with nanocomposite materials. The findings of the salt spray showed that the steel substrate painted with the primer based on bio CuO/ZnO and bio ZnO nanocomposite modified PEA and alkyd resin had excellent adhesion. This was seen in the PEA and alkyd modified with nanocomposite coatings that performed better after 450 h of exposure to the salt spray environment compared with the sample blank. For every coating exposure time, coating performance was continuously increased. Relationships between coating characteristics and performance can be credited with this. In this regard, the primary cause of coating failure is the substrate's adhesion to the coat. Strong adhesion also stops moisture vapor from penetrating the coating and condensing in an area with low adhesion, which would otherwise cause the coating to blister. This is especially important when choosing the appropriate coating systems. In this work, it was observed that increasing the amount of SDEA in the modified PEA and alkyd resins and the hydroxyl contents of cured PEA and alkyd resins also increased the adhesion of the coat to steel. It has been shown that the nonmodified resins and nanocomposites with the PEA and alkyd have a non-positive effect on the adhesion of the alkyd coating to the steel metal. So, it was affected when exposed to the salt spray^33^. Hyperbranched alkyd matrices have stronger anticorrosive protection than regular matrices, according to prior investigations^58^. A traditional alkyd matrix was prepared with reduced resistance to NaOH solution, and the manufactured coating layers were totally separated^60^. According to the tabulated results in Table 6, the corrosion spread of steel-painted panels based on unmodified PEA and alkyd was greater than that of PEA nanocomposite CuO/ZnO and nanocomposite bio ZnO alkyd, which was approximately 1.5–2 mm; in the case of steel painted panels based on PEA nanocomposite bio CuO/ZnO, the corrosion resistance was higher than that of nanocomposite bio ZnO alkyd. This finding reflects the effects of the distribution of the bio nano CuO/ZnO and bio ZnO nanocomposite modified alkyd on corrosion protection. The gradual insertion of nano-CuO/ZnO and also ZnO decreases the density of blisters and the rusting of spots compared with the painted steel panel based on unmodified PEA and alkyd. From the obtained results of the corrosion resistance, we can conclude that the improvements may be achieved by evenly dispersing nanoparticles inside the polymer matrix, resulting in improved adhesion to the substrate^62^. And the introduction of the nitrogen element into the PEA structure Also, the protection of mild steel from corrosion is attributed to its being impermeable to water and corrosive ions and having reduced water permeability^25^**.**

**Table 6:** Evaluation of corrosion resistance of coated steel panels composed of bio ZnO nanocomposite modified poly (ester amide) resin after exposure to salt spray for 450 h.

Sample ID	Degree of rusting	Blistering		Scribe failure mm
		Size	Frequency	
Sample blank withoud modictaion	5	6	F	5
Coating based on bio ZnO nanocomposite poly(ester-amide) resin	8	8	Medium dense	9
Coating based on bio CuO/ZnO nanocomposite poly(ester-amide) resin	9	9	Medium dense	9

#### Mechanism of corrosion protection

The mechanism of protecting bio ZnO, CuO/ZnO nanocomposite PEA and alkyd coated mild steel substrate against corrosion through electrostatic repulsion of chloride species is shown in the schematic in Fig. 13. It is depicted in this schematic that after making cross-cut along with the coatings, created microcavities on the coating surfaces when exposed to saline and foggy environment for better understanding of the corrosion inhibition property during the corrosion test of the unmodified and modified nanocomposite PEA and alkyd coating. These cavities allowed the ingress of the corrosive solution to the coating-metal interface. In the bio ZnO, CuO/ZnO nanocomposite PEA and alkyd coatings, chloride ions were restricted by bio ZnO, CuO/ZnO nanocomposite PEA and alkyd through electrostatic repulsion, comparing with the coated steel panel based on unmodified alkyd and PEA resins, which did not hinder or restrict the ingress of neutral species (oxygen and water) through the cavities on the coating surface. Over time, oxygen and water diffused into the coating-metal interface to initiate uniform corrosion by the formation of ferrous hydroxide as follows. This repulsion of chlorides prevented chloride-initiated corrosion at the coating-metal interface as shown in Fig. 14^63^.

1 $${\text{Fe}} + \frac{1}{2}{\text{ O}}_{{2}} + {\text{ H}}_{{2}} {\text{O }} \to {\text{ Fe}}\left( {{\text{OH}}} \right)_{{2}}$$

With more oxygen diffusion into the coating, ferrous hydroxide is converted to ferric oxide, indicated as deep brown in schematic (a). This conversion can be described thus:

2 $${\text{3Fe}}\left( {{\text{OH}}} \right)_{{2}} + {\text{ O}}_{{2}} \to {\text{ Fe}}_{{3}} {\text{O}}_{{4}} + {\text{ 3H}}_{{2}} {\text{O}}$$

However, in schematic Fig. 13B and C, the mild steel-coated bio nanocomposite coating samples exhibited severe pitting attacks following weak electrostatic repulsion of the chloride ions. Unlike the unmodified coated samples. Hence, the repulsion of chloride ions was ineffective for the coated sample based on nanocomposite-modified alkyd and PEA, compared to the unmodified coatings, leading to corrosion at the coating-metal interface of the coated sample based on unmodified alkyd and PEA Fig. 13A.

The pits initiation by chloride ions at the interface starts with the formation of ferrous cations according to the following two reactions^64^:

3 $${\text{Fe }} + {\text{ Cl}}^{ - } = {\text{ FeCl}}^{ - }_{{{\text{interface}}}}$$

The presence of water and oxygen causes the oxidation of iron to ferrous hydroxide,

4 $${\text{Fe }} + {\text{ O}}_{{2}} + {\text{ H}}_{{2}} {\text{O }} \to {\text{ Fe}}\left( {{\text{OH}}} \right)_{{\text{2 interface}}}$$

The ferrous hydroxide further combined with the ferrous chloride as the following:

5 $${\text{Fe}}\left( {{\text{OH}}} \right)_{{{\text{interface}}}} + {\text{FeCl}}^{ - }_{{{\text{interface}}}} = {\text{Fe }} + {\text{FeOH}}^{ + } + {\text{Cl}}^{ - } + {\text{2e}}^{ - }$$

6 $${\text{FeOH}}^{ + } + {\text{ H}}^{ + } = {\text{ Fe}}^{{{2} + }}_{{{\text{interface}}}} + {\text{ H}}_{{2}} {\text{O}}$$

Moreover, the improvement of anticorrosion performance using ZnO, CuO/ZnO nanocomposite coating might be ascribed to the following reasons: (1) the alkyd and poly(ester-amide) coatings might be regarded as a physical barrier layer, (2) the nanoscale ZnO, CuO/ZnO could effectively form a dense passivation film with Fe element, which significantly prevented the corrosive mediums such as water, oxygen, chloride ion from further diffusing into the surface of the mild metal. This was why ZnO, CuO/ZnO nanocomposite coating owned the best anti-corrosion performance compared to other nanocomposite coatings (A,) (blank coating), (3) the nanosheet structure of ZnO, CuO/ZnO nanocomposite coating provided an extra barrier film to significantly prevent the water, oxygen, chloride ion from penetrating the pores, thereby preventing the corrosion of the underlying metal, and (4) due to, the well-dispersed ZnO, CuO/ZnO hybrids in alkyd and poly(ester-amide) could enhance the corrosion protection performance of the coatings (5) may be also the improvement due to mixed metal oxide NPs which are composite with highly rich nitrogen-modified binders that provide corrosion protection and prevent anodic corrosion by forming a protective passive layer on the substrate by the delocalized electrons on the nitrogen atoms in addition to the presence of metal nanoparticles^58^. It has been further reported that the modified alkyd and PEA coatings act as an anticorrosive binder e to retain the metal in the passive state^35,44,45^. Further mixed metal oxide nanocomposite such as TiO_2_, Al_2_O_3_, Mn, Co, Cd, Cu and Zn^65–68^ alkyd and PEA act as a mediator for anodic current between the passivated mild steel surface and oxygen reduction in the coating. The Fe^2+^ formed due to the initial corrosion reaction reacts with the dopant anions generated from nanocomposite alkyd and PEA reduction leading to the formation of insoluble iron-dopant salt which further passivates the mild steel surface along with nanocomposite alkyd and PEA.

#### SEM of the corrosion resistance of painted steel panels after corrosion

**Figure 15:**
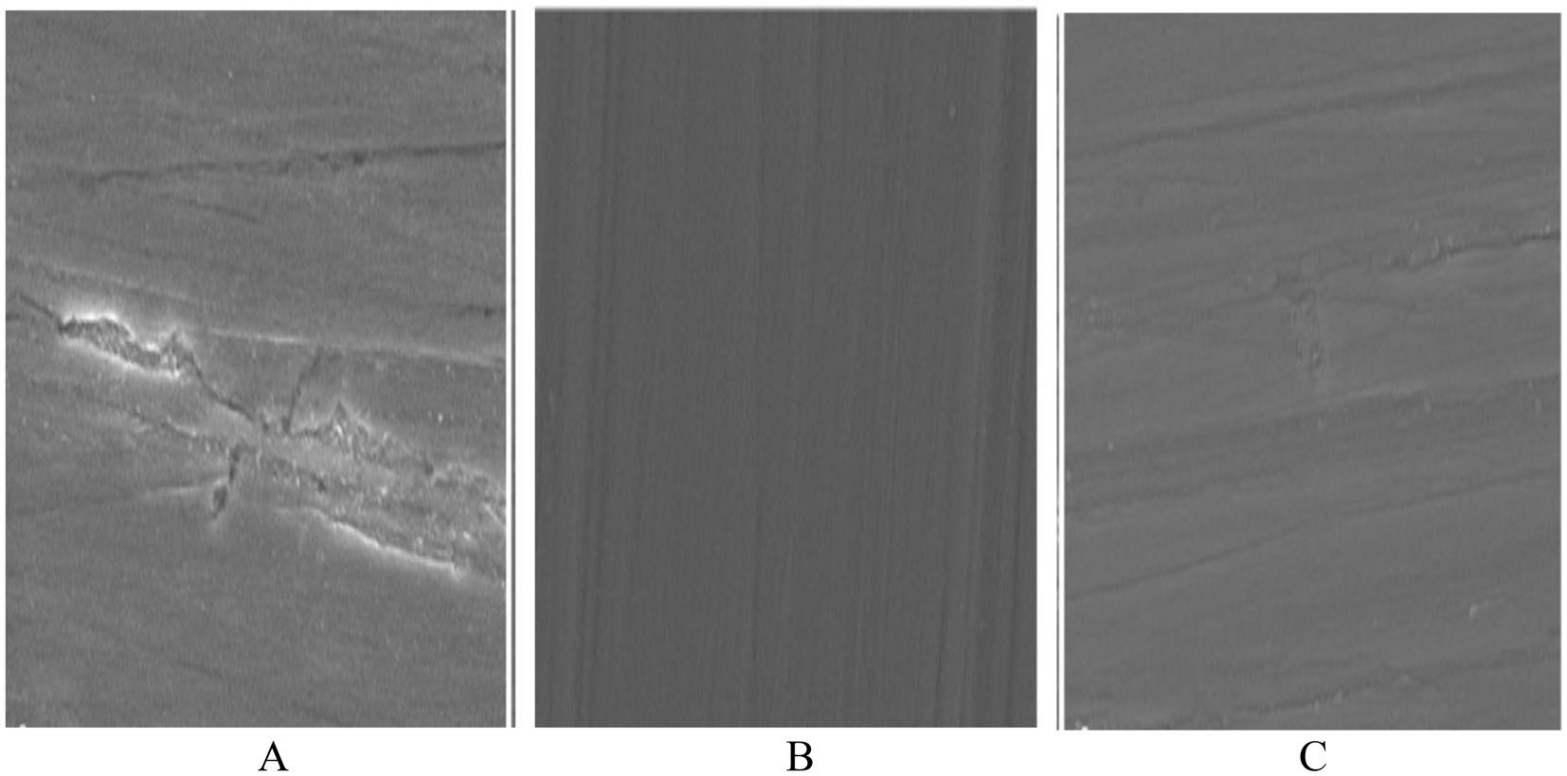
SEM images of (**A**) blank coating, (**B**) coating based on CuO/ZnO nanocompos-ite modified PEA resin (**C**) coating based on bio ZnO nanocomposite modified alkyd resin.

The SEM micrograph, as shown in Fig. 15 of the coated steel panels After corrosion shows a few holes and localized corrosion is apparent, especially for the valleys and defect areas for blank coated steel panels, maybe this is attributed to the presence of agglomeration in the blank coating formulation, which may cause the differences in the coating’s surface structure and composition by which the localized corrosion of coatings not based on nanocomposite PEA and alkyd can be induced. While the surfaces of coated steel panels based on CuO/ZnO nanocomposite PEA and coated steel panels based on bio ZnO nanocomposite alkyd are smooth and nearly no obvious holes can be seen, the surface is much smoother than the surface of the blank coating, and this is due to the same reasons mentioned previously, such as the presence of nitrogen elements, and the prepared nanocomposite modified PEA and nanocomposite modified alkyd resin.

## Conclusion

In this study, a successful preparation of two new modified PEA and alkyd resins based on sunflower oil fatty acid was achieved. The modification has been done by partial replacement of monoglyceride with salicylic diethanolamine as the ingredient source of polyol. The FTIR and ^1^HNMR spectra confirmed the chemical structures of both the new polyol and the resins with the functional groups present. The green nano-biosynthesis method was employed to prepare ZnO and CuO/ZnO nanoparticles, which were then incorporated in the modified PEA and alkyd resins. The nanocomposites obtained were characterized by FT-IR, TGA, and SEM. Then, the prepared nanocomposite resins were formulated as binders in paint formulation to be evaluated as anticorrosive resins for steel protection. It was observed that the modified resins, either modified PEA or modified alkyd resins, showed great improvement in overall coating properties compared to other unmodified resins. The existence of salicylic diethanolamine as a modifier led to significantly improved chemical resistance and physio-mechanical properties such as scratch hardness, gloss, adhesion, and thermal stability, but it was noticed that in the case of coated films based on modified PEA, these properties were improved more than those based on modified alkyd and this may be due to the presence of nitrogen elements in the structure of the polymer. Modified nanocomposite binders showed better film performance in terms of corrosion when blended within the paint formulation. The anticorrosive results of the formulated coating revealed that the film-loaded, well-dispersed bio-ZnO and CuO/ZnO spherical nanofillers had a perfect anticorrosive effect when applied for steel protection.

## Supplementary Information

Supplementary Figures.

## Data Availability

The datasets used and analyzed during the current study are available from the corresponding author upon reasonable request.
