# Supplementary material for: Preparation of new surface coating based on modified oil-based polymers blended with ZnO and CuZnO NPs for steel protection
Source: Sci Rep. 2023 May 4;13:7268. doi: 10.1038/s41598-023-34085-z (PMC10160130; doi:10.1038/s41598-023-34085-z)
Supplement: Supplementary file 1 — Supplementary Figures. [file 41598_2023_34085_MOESM1_ESM.docx]

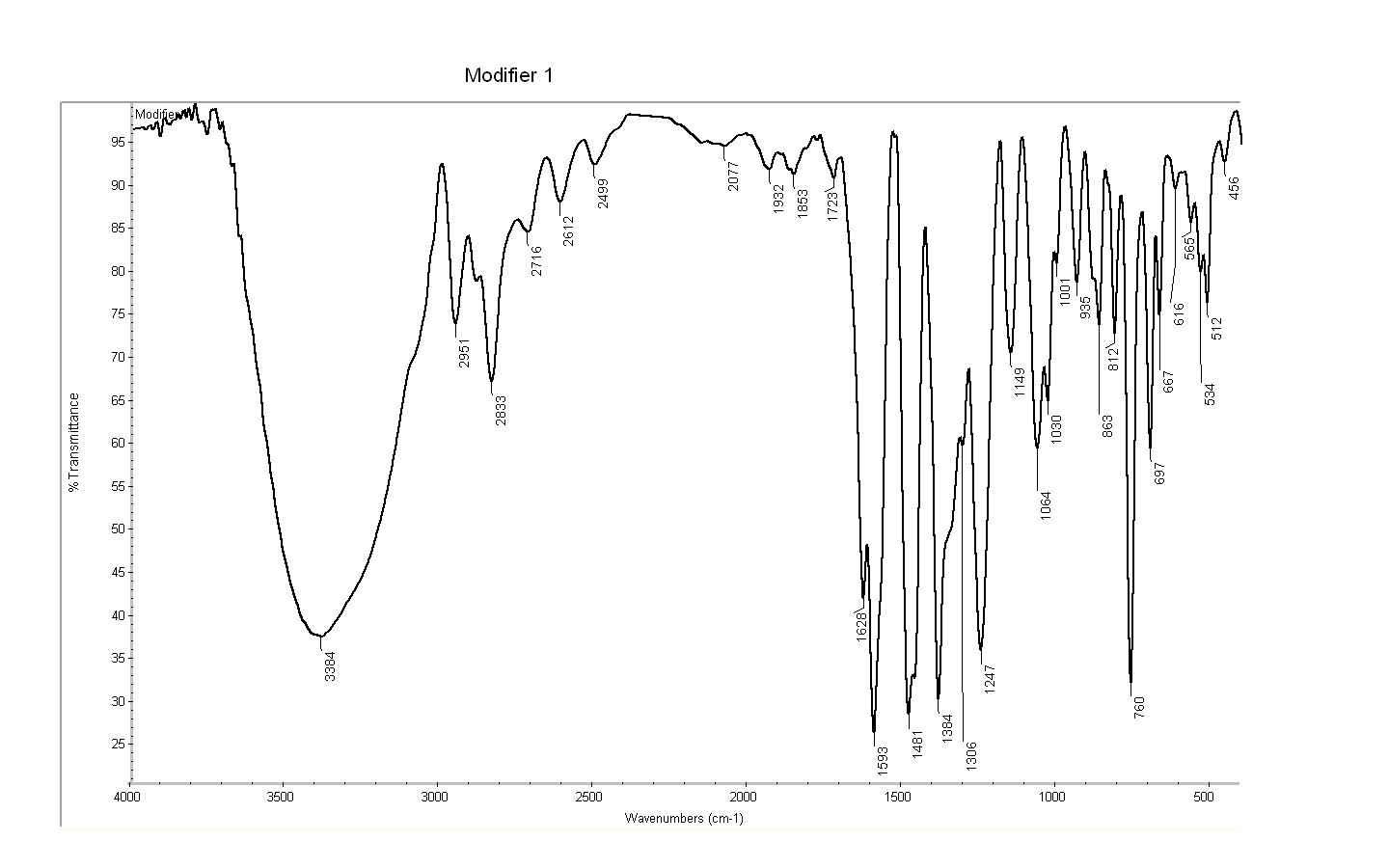
**S (1). FT-IR spectra of the prepared Salicylic diethanolamine [SDEA].**


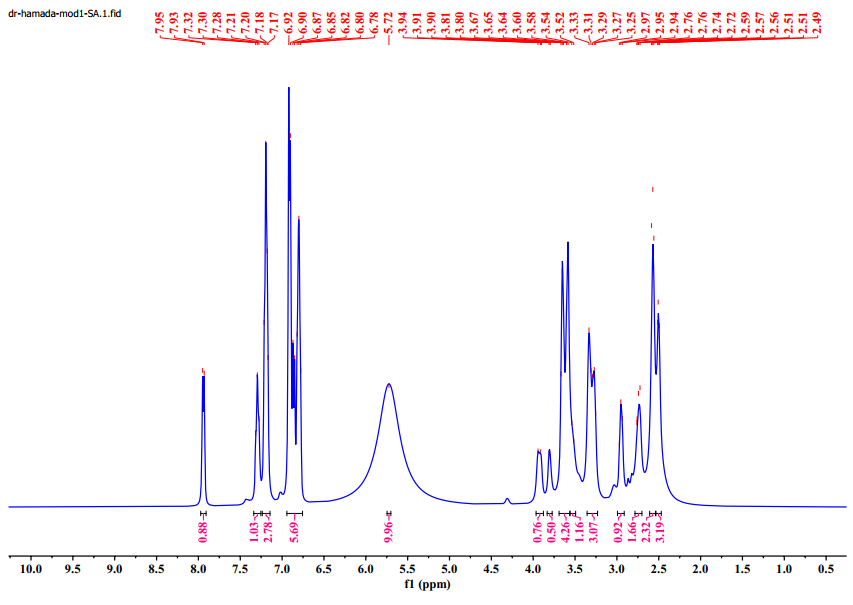


**S (2). H-NMR spectra of the prepared Salicylic diethanolamine [SDEA].**


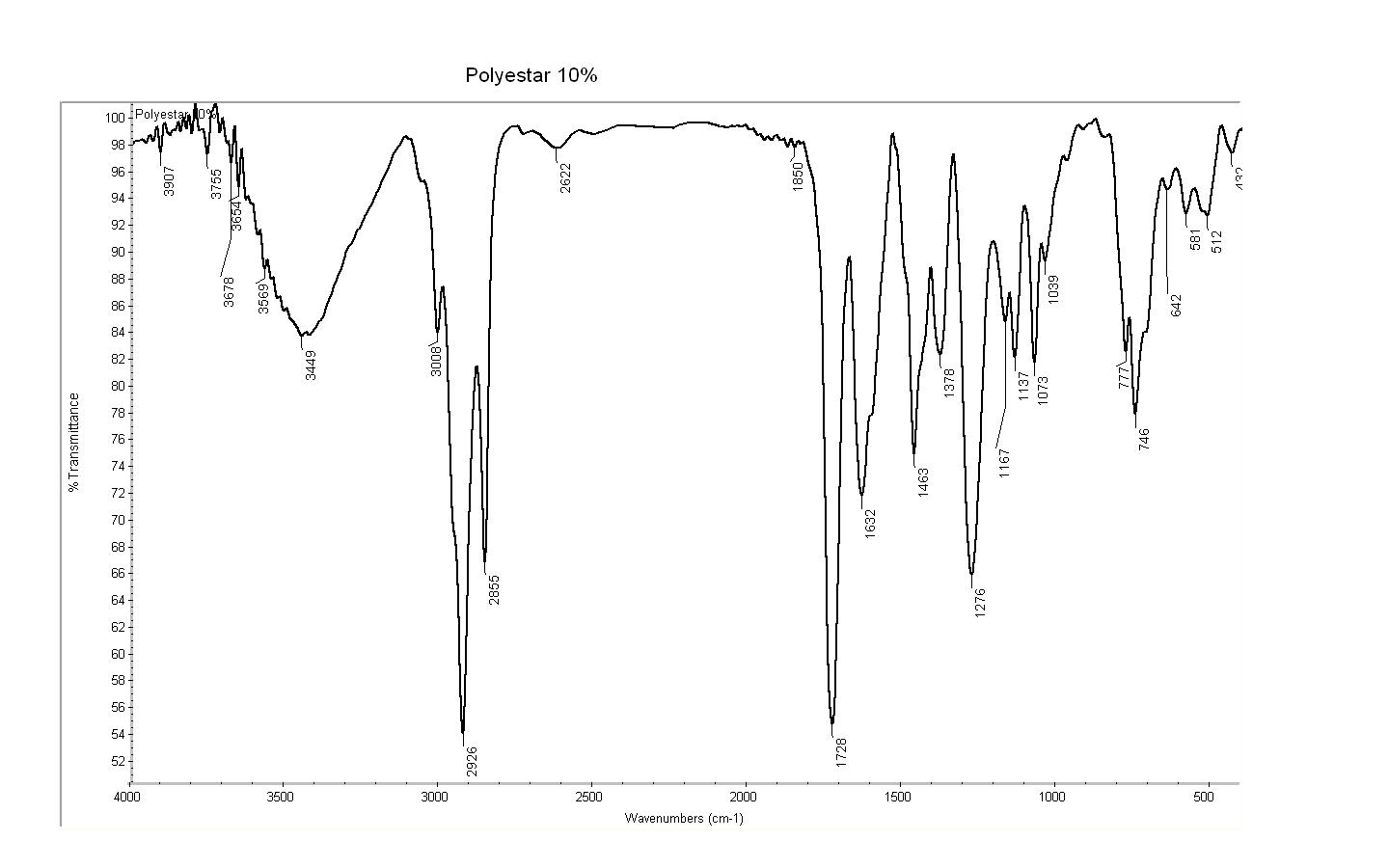


**S (3). FT-IR spectroscopy SDEA modified poly(ester-amide) resin (PEA)**

**
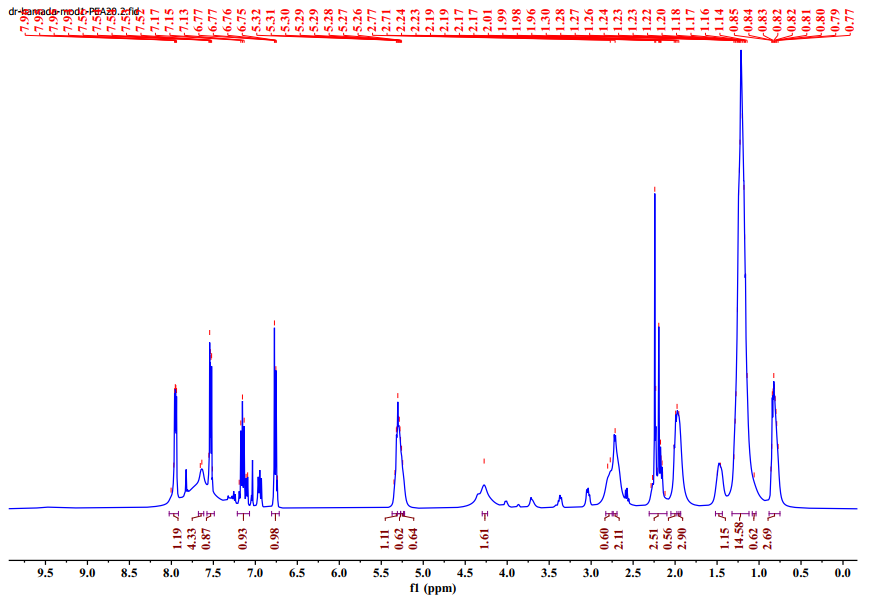
**

**S (4). H-NMR spectroscopy SDEA modified poly(ester-amide) resin (PEA)**


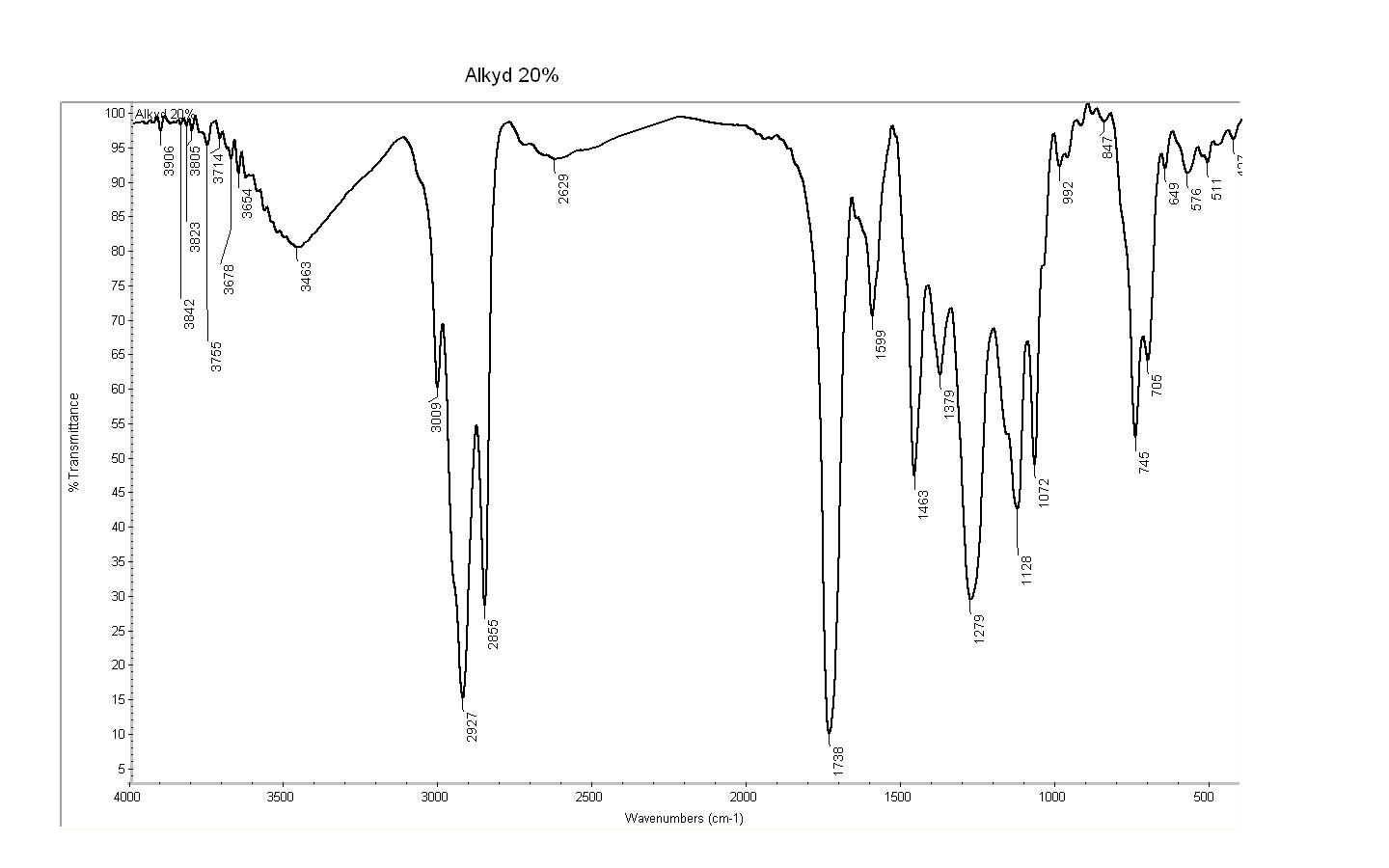


**S (5). FT-IR spectroscopy SDEA modified alkyd resin**

**S (6). H-NMR spectroscopy SDEA modified alkyd resin (PEA)**

**
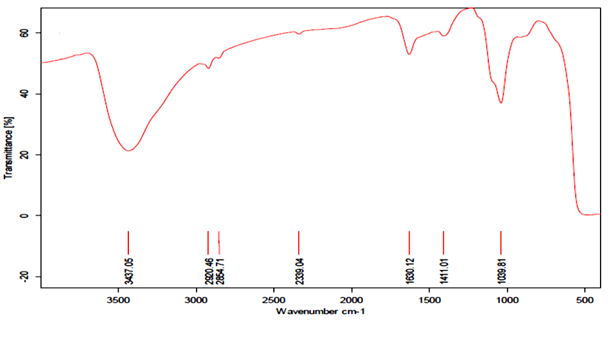
**

**S (7). FTIR spectra of the synthesized ZnO nanoparticles**

**
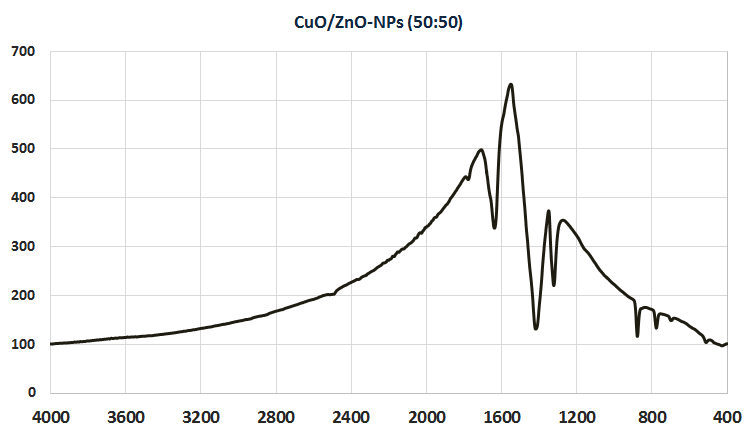
**

**S (8)** FT-IR spectra of the biomass ﬁltrate of the *P. corylophilum* As-1, CuO / ZnO NPs

¼


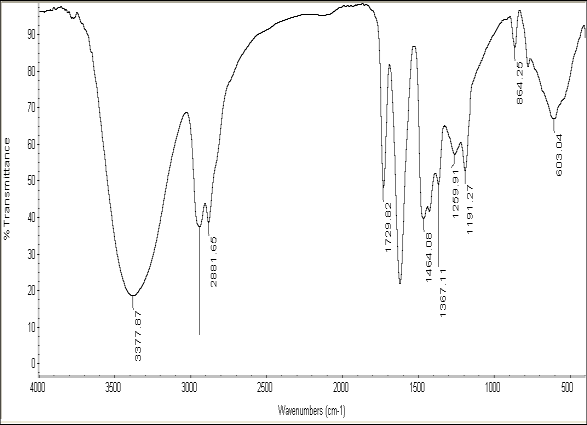


**S (9). FT-IR spectroscopy bio ZnO nanocomposite modified alkyd resin (PEA)**
